# Analysis of spiking synchrony in visual cortex reveals distinct types of top-down modulation signals for spatial and object-based attention

**DOI:** 10.1371/journal.pcbi.1008829

**Published:** 2021-03-25

**Authors:** Nobuhiko Wagatsuma, Brian Hu, Rüdiger von der Heydt, Ernst Niebur

**Affiliations:** 1 Faculty of Science, Toho University, Funabashi, Chiba, Japan; 2 Allen Institute for Brain Science, Seattle, Washington, United States of America; 3 Zanvyl Krieger Mind/Brain Institute, and Solomon Snyder Department of Neuroscience, Johns Hopkins University, Baltimore, Maryland, United States of America; Dartmouth College, UNITED STATES

## Abstract

The activity of a border ownership selective (BOS) neuron indicates where a foreground object is located relative to its (classical) receptive field (RF). A population of BOS neurons thus provides an important component of perceptual grouping, the organization of the visual scene into objects. In previous theoretical work, it has been suggested that this grouping mechanism is implemented by a population of dedicated grouping (“*G*”) cells that integrate the activity of the distributed feature cells representing an object and, by feedback, modulate the same cells, thus making them border ownership selective. The feedback modulation by *G* cells is thought to also provide the mechanism for object-based attention. A recent modeling study showed that modulatory common feedback, implemented by synapses with N-methyl-D-aspartate (NMDA)-type glutamate receptors, accounts for the experimentally observed synchrony in spike trains of BOS neurons and the shape of cross-correlations between them, including its dependence on the attentional state. However, that study was limited to pairs of BOS neurons with consistent border ownership preferences, defined as two neurons tuned to respond to the same visual object, in which attention decreases synchrony. But attention has also been shown to increase synchrony in neurons with inconsistent border ownership selectivity. Here we extend the computational model from the previous study to fully understand these effects of attention. We postulate the existence of a second type of *G*-cell that represents spatial attention by modulating the activity of all BOS cells in a spatially defined area. Simulations of this model show that a combination of spatial and object-based mechanisms fully accounts for the observed pattern of synchrony between BOS neurons. Our results suggest that modulatory feedback from *G*-cells may underlie both spatial and object-based attention.

## Author summary

Vision allows us to make sense out of a very complex signal, the patterns of light rays reaching our eyes. Two mechanisms are essential for this: perceptual organization which structures the input into meaningful visual objects, and attention which selects only the most important parts in the input. Prior work suggests that both of these mechanisms are implemented by neurons called grouping cells. These organize the object features into coherent entities (perceptual grouping) and access them as needed (selective attention). For technical reasons it is difficult to observe grouping cells but their effect can be seen in the influence they have on responses of other classes of cells. These responses have been measured experimentally and it was found that they depend in unexpected ways on where the subject was attending. Using a computational model, we here demonstrate that the responses can be understood in terms of the interaction between two kinds of selective attention, both of which are known to occur in primate perception. One is attention to a specific area in the environment, the other is to specific objects. A model including both of these attentional mechanisms generates neuronal responses in agreement with the observed patterns of neural activity.

## Introduction

In this study, we focus on the interplay of two features of intermediate vision. The first is selective attention which enhances perception of particular sensory stimuli [1–5]. Top-down visual attention can be categorized into at least three distinct types: spatial, feature-based, and object-based attention [6–8]. In this study, we focus on spatial and object-based attention. It has been suggested that these types of attention rely on distinct cortical pathways [9] for enhancing the related neural responses and for improving the discriminability of visual stimuli [10–12].

The second feature is figure-ground segregation, the integration of visual features into objects and the segmentation between different objects and the background. This is an important step in understanding complex scenes, with images of many objects projected simultaneously onto the retinae. It has been proposed that an early step in figure-ground segregation is to establish on which side of the border of a foreground object (figure) this object is located. This has been called border ownership since the foreground object, which is closer to the observer, determines the fate of the border (*e.g*. when the object is moving) and therefore “owns” it [13–15].

The majority of neurons in intermediate-level visual area V2 are border ownership selective, with their responses depending on which side of a border owns the border. These are the Border Ownership Selective (BOS) neurons [16]. Various characteristics and mechanisms of BOS neurons have been investigated through physiological methods [17–20], studies of human perception [21–23] and computational models [24–31]. One computational method that is designed to draw conclusions on the structure of the neuronal circuitry underlying the observed activity patterns is the analysis of neuronal correlations. In particular, common input plays a critical role for inducing synchronized responses between postsynaptic neurons. For this reason, it is believed that analyses of spike train correlations and spike synchrony between neurons can provide insights into neuronal connectivity [32–34] (but see ref [35] for a cautionary note). Intriguingly, several studies have indicated that common input may modulate the activities of postsynaptic neurons rather than driving it, *i.e*. increase its mean firing rate by itself. For example, modulatory input from higher visual areas increases the firing rates of striate (V1) and extrastriate (V2) neurons [25, 36] related to top-down attention [37] and figure-ground segregation [16, 38]. In this case, common input spikes from a higher area may not evoke action potentials in the target neurons by themselves but will transiently enhance the firing rate increase that is caused by feedforward input from bottom-up visual stimuli.

**Fig 1:**
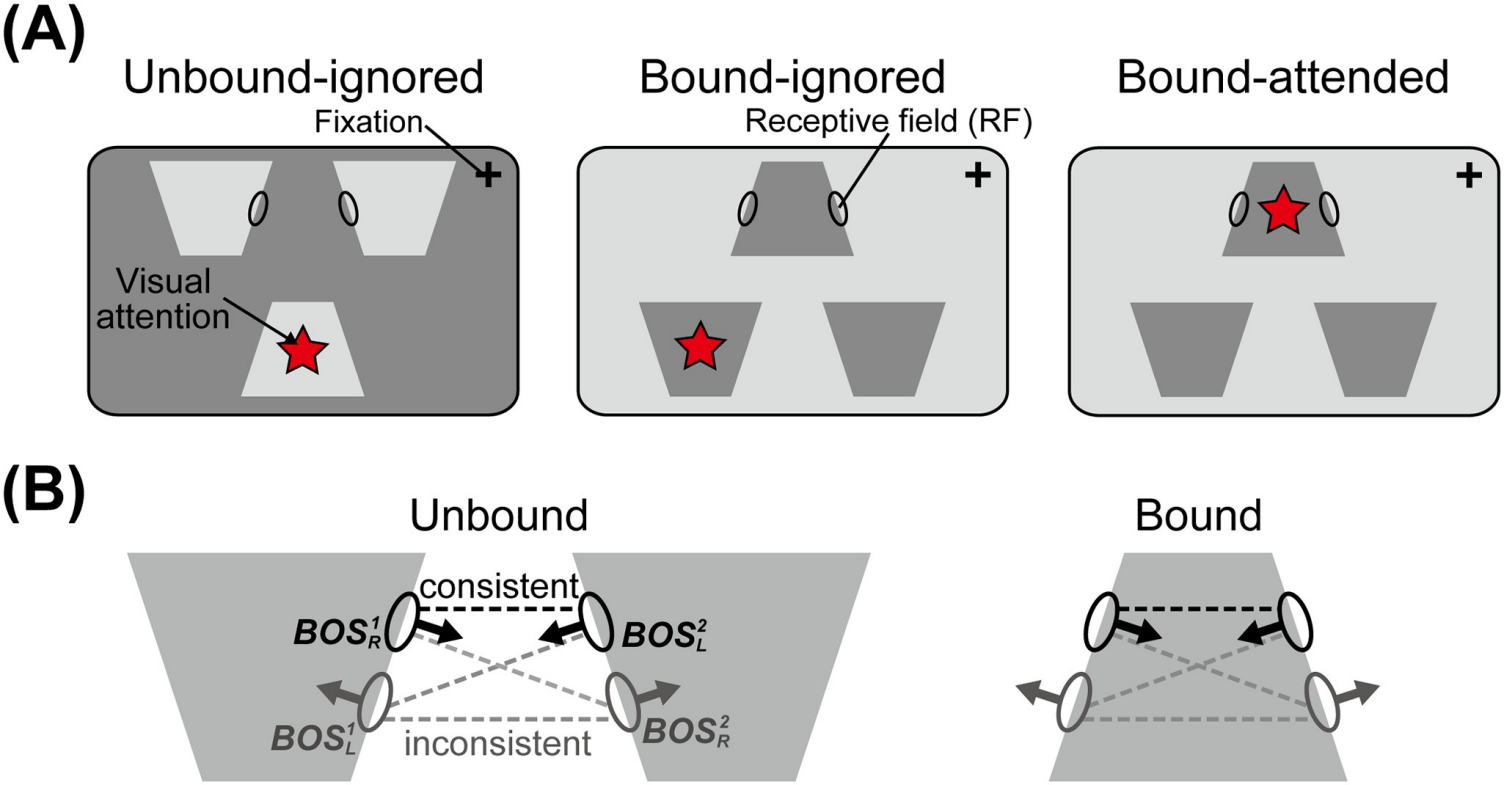
Pairs of BOS neurons and conditions for visual input and attention (modified from ref [39]). A: Stimulus displays for testing the effects of object integration and selective attention. Ellipses on the borders of the keystone-like objects represent the receptive fields (RFs) of border ownership selective (BOS) neurons. In each display, three separate objects are presented. In the left display the RFs of two neurons are on the borders of two *different* objects (“unbound” condition). In the middle and right displays the two RFs lie on the borders of the *same* object (“bound” condition). Note that the visual stimuli in and around the RFs are identical in all three conditions, but represent parts of two separate objects in the left display and parts of the same object in the other two displays. In these experiments, the monkey attended one of the objects, as shown by a red star (not part of the display). Such an object is called “attended” while objects that are not attended are referred to as “ignored.” B: Consistent and inconsistent pairs. Arrows from an RF point toward the preferred side of the corresponding BOS neuron. Subscripts *L* and *R* indicate left and right side-of-figure preferences, respectively, while retinotopic position is represented by the superscripts “1” and “2”. RFs of neurons whose border ownership preferences are consistent with representing a common object are connected by black dashed lines (“consistent pairs”), while RFs of neurons with inconsistent preferences are connected by gray dashed lines (“inconsistent pairs”).

Recently Martin and von der Heydt [39] have physiologically characterized effects of grouping structure and attention on spike train correlation between BOS neurons (Fig 1). Building on previous work [18] that failed to support the “binding-by-synchrony” hypothesis (review: ref [40]), their work showed that spiking synchrony between neurons depends on their border ownership selectivity. For pairs of neurons with consistent border ownership preference, stimulation by a common object increased spiking synchrony, but selective attention to the object, while increasing firing rates, decreased spiking synchrony. A recently proposed computational model explains both the firing rate changes and synchrony structure in these neurons [41]. The model is based on the assumption that feedback from hypothetical grouping cells (*G*-cells) at higher visual areas modulates the activities of BOS neurons. Importantly, the feedback does not drive the activity of neurons by itself but rather shapes the activity caused by visual input. The model postulates that this modulatory feedback is implemented by glutamatergic synapses of the N-methyl-D-aspartate (NMDA) type [42, 43]. Activation of NMDA receptors by itself does not increase firing rates of postsynaptic neurons substantially, but it increases the effect of excitatory input from other types of receptors, typically of the glutamatergic *α*-amino-3-hydroxy-5-methyl-4-isoxazolepropionic acid (AMPA) type.

A second major result of the study by Martin and von der Heydt [39] is that for pairs of neurons with *inconsistent* BOS preference, attention to the common object increased spike synchrony. This observation was not addressed in the computational model proposed in ref. [41] which focused strictly on consistent neuronal pairs and the paradoxical reduction of synchrony by attention. The main goal of the present study is to understand why attention can increase synchrony between inconsistent pairs.

**Fig 2:**
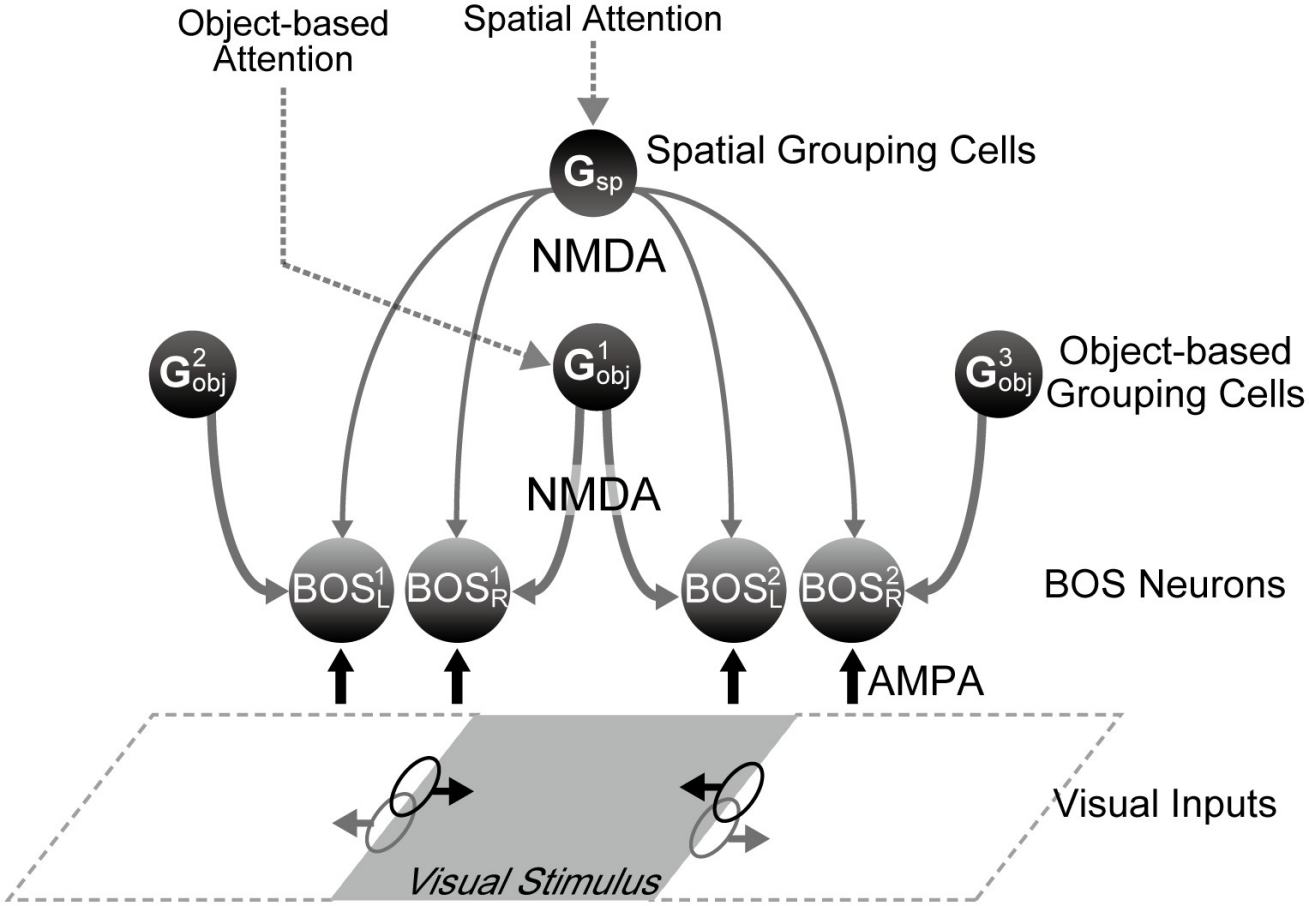
Model architecture. Two different types of *G*-cells (balls with “G”) are part of the model: spatial grouping cells (*G*_*sp*_) and object-based grouping cells (*G*_*obj*_). Whereas *G*_*sp*_-cells implement spatial attention, *i.e*. attention to everything within a circumscribed spatially defined area, *G*_*obj*_-cells impart grouping structure of objects in the scene and mediate object-based attention. The latter are similar to the grouping cells from refs [25, 26, 41]. Feedback signals from these *G*-cells modulate activity of BOS neurons (balls with “BOS”) by NMDA-type connections (gray downward pointing arrows). Black and gray ellipses represent the locations of RFs of BOS neurons which are driven by visual input through AMPA-type synapses (black upwards pointing arrows). Black and gray horizontal arrows from RFs point toward the preferred side of the corresponding BOS neuron. For description of subscripts and superscripts of BOS cells see text.

As in previous models, we assume that responses of model BOS neurons are modulated by feedback from *G*-cells which organize the responses of BOS neurons and mediate top-down attention [25, 26, 28]. Going beyond earlier work, we assume two distinct classes of *G*-cells, one responsible for spatial attention and one for attention to objects (Fig 2). Both classes of *G*-cells provide modulatory feedback to BOS neurons via NMDA synapses. Self et al. [42] have reported that feedforward input to V1 is mainly provided by AMPA type synaptic currents whereas feedback signals mediated by NMDA synaptic receptor underlie figure-ground modulation. Herrero *et al*. [43] showed the importance of NMDA receptors for mediating the feedback signals including selective attention. Simulations of the proposed model indicate overall agreement with responses of BOS neurons as reported by Martin and von der Heydt [39]. These results suggest that feedback signals modulate the responses of feature selective neurons in lower-level visual areas, and that there are two types of feedback signals, one that serves spatial attention, and another that facilitates object-based attention.

In this study we hypothesize, and we support this hypothesis by computational studies, that the feedback signals are responsible for the neurophysiologically observed correlation structure between BOS neurons. All BOS neurons that represent different parts of the same perceptual object receive input from those G cells that represent this object. This common input generates correlation between their spike trains. The situation becomes more complicated, however, when attention is taken into account. We showed in our previous study [41] the non-monotonic effect of G-cell firing rates on synchrony between border ownership selective (BOS) cells (see Fig 4 of [41]). The observed lower correlation between BOS neurons representing parts of the same object when this object is attended compared to when it is not attended could then be understood (see Fig 3 of [41]) since the effects of both grouping and attention to objects modify the firing rates of BOS cells. That study did, however, not deal with inconsistent BOS cell pairs. In the present report we show that increased synchrony due to attention can be explained as a consequence of spatial attention, implemented in a separate class of G cells.

## Results

### Model structure

To investigate the neuronal mechanisms of border ownership selectivity in visual cortex, we study the behavior of our model (Fig 2) with simulated versions of visual input and a simple model for the animal’s attentional state. Border ownership relations are represented by BOS neurons, four of which ($BOS_{R}^{1}$, $BOS_{L}^{1}$, $BOS_{R}^{2}$, $BOS_{L}^{2}$) are shown in Fig 2. Their subscripts L and R indicate their left and right side-of-figure preferences for the stimulus shown, the gray parallelogram. Their retinotopic position is denoted by the superscript “1” for the receptive fields (RFs) in the left side of the visual field (left ellipses) and “2” for the right side (right ellipses).

Per the definition in Fig 1B, $BOS_{R}^{1}$ and $BOS_{L}^{2}$ neurons form a consistent pair, all other neuron pairs are inconsistent. BOS neurons integrate bottom-up input originating in visual stimuli and mediated by AMPA-type synapses with top-down modulatory feedback mediated by NMDA-type synapses. Bottom-up inputs representing visual stimuli to each BOS neuron are given by independent stochastic processes with Poisson statistics. In our model, the activity of *G*-cells represents both the stimulus configuration as well as the attentional state of the animal. We assume two types of grouping cells as the source of top-down feedback signals. One type represents object-based grouping (*G*_*obj*_) cells, which impart the grouping structure and mediate object-based attention. The other type consists of spatial grouping (*G*_*sp*_) cells, which implement attention to a purely spatially defined region of the visual field, independent of its contents. In the example configuration of Fig 2, whereas feedback from $G_{obj}^{1}$ cells functions as the common inputs to the $BOS_{R}^{1}$ and $BOS_{L}^{2}$ neurons, representing the same object (and therefore making the latter a consistent pair), signals from $G_{obj}^{2}$ and $G_{obj}^{3}$ are independently given to $BOS_{L}^{1}$ and $BOS_{R}^{2}$ respectively. Additionally, the *G*_*sp*_ cell activates all BOS neurons homogeneously, irrespective of their RF location and their border ownership selectivity.

All results of our study are formulated in terms of changes of mean firing rates of BOS neurons and of the spike-spike correlation functions of pairs of BOS neurons. Any pair of BOS neurons can be in one of four possible states with respect to the activating visual object(s). These four states are the combinations of two types, bound *vs*. unbound, and attended *vs*. not attended. For the first state, the two members of the BOS neuron pair can represent parts of the same object, or of different objects. In the first case, the neurons participate in the representation of an integrated, or bound, object. In the second case, they represent parts of different objects which are not bound to a coherent object. For simplicity, we call this an “unbound” situation, see Fig 1. The second state regards attention. Attention may be either on an object with some of its parts in the RF of one or both of the neurons, or attention can be at a position elsewhere in the visual field, far from the RFs of the considered neurons. In the first case, attention is on the BOS neuron(s) while in the second case both are ignored. These two binary types of states (attended *vs*. ignored, bound *vs*. unbound) are considered independent of each other, therefore there are four possible combinations. We do not consider one of these conditions, unbound-attended, since we are not aware of any neurophysiological experiments addressing situations in which attention is directed towards disconnected visual features.

In our model, the attentional state is represented by the activity level of *G* cells. We assume that *G* cells representing an attended object have a higher firing rate than those representing an ignored object, see Table 1. Our model does not describe how these activity levels derive, there are many models of the control of selective attention that describe possible mechanisms, *e.g*. [44] for bottom-up attention or ref [45] for top-down attention.

**Table 1:** Firing rates of model *G*-cells in different stimulus and attention conditions. The labels in the first column refer to the stimulus and attention conditions in the RF of $G_{obj}^{1}$. Numbers in other columns are firing rates of *G* cells listed in the first row of the respective column, under conditions in the RF of $G_{obj}^{1}$ that are described in the first column.

Stimulus in $G_{obj}^{1}$ RF	$G_{obj}^{1}$	$G_{obj}^{2}$	$G_{obj}^{3}$	*G*_*sp*_
**Unbound-ignored**	5Hz	30Hz	30Hz	3Hz
**Bound-ignored**	30Hz	5Hz	5Hz	3Hz
**Bound-attended**	60Hz	2.5Hz	2.5Hz	15Hz

Likewise, we assume that a *G* cell has a higher firing rate in the bound condition than in the unbound condition, because in the bound condition it integrates features all around the boundary of the object, each of which is represented by the activity of individual BOS neurons, whereas in the unbound condition, it integrates only two edges, each from one of the lateral objects (dashed outlines in Fig 2). Also the two grouping cells which would represent the lateral objects, ${\text{G}}_{obj}^{3}$ and ${\text{G}}_{obj}^{3}$ would each receive input from BOS cells of only one edge, ${\text{BOS}}_{L}^{1}$ and ${\text{BOS}}_{R}^{2}$, respectively. Therefore, the *G* cell in the bound condition receives more bottom-up input and, as a result, has a higher mean firing rate.

Mechanisms leading to increased *G* cell activity are quantitatively described in several published computational studies [25, 26, 28–30, 46]. While the argument presented so far applies only to isolated objects, new experimental results [47] show that also *G* cells representing foreground objects in cluttered scenes likely have substantially higher firing rates than *G* cells representing the partially obscured objects, see the section on cluttered scenes in the Discussion. In this study, we therefore assume that differential activity levels (firing rates) are present for bound (higher rates) and unbound conditions (lower rates), without explicitly implementing how these differences arise.

The firing rates of model *G*-cells for all three stimulus and attention conditions are summarized in Table 1 and in the Materials and methods section. For cell $G_{obj}^{1}$, the situation shown in Fig 2 and in the middle and right panels of Fig 1A illustrates the bound condition. As seen in column ‘$G_{obj}^{1}$’ of the Table 1, its firing rate is 30Hz if the object is ignored (middle panel of Fig 1A) and 60Hz if it is attended (right panel of Fig 1A) since we assume that the firing rate effects of binding and attention are cumulative. In the unbound-ignored condition it fires with a low spontaneous rate of 5Hz.

Columns ‘$G_{obj}^{2}$’ and ‘$G_{obj}^{3}$’ of Table 1 show the firing rates of the *G* cells representing objects left and right of $G_{obj}^{1}$, respectively, in the geometrical situation of Fig 2. In the Unbound-ignored condition, each of these cells has an (ignored) object in its RF (left panel of Fig 1A). This is the same situation as $G_{obj}^{1}$ finds itself in the Bound-ignored condition and the same firing rates apply, 30Hz. In contrast, in the Bound-ignored condition (center panel of Fig 1A) there is no object in the RF of these cells, the same situation as $G_{obj}^{1}$ finds itself in the Unbound-ignored condition, and the firing rate is the same (5Hz). In the Bound-attended condition (right panel in Fig 1A) there is no object in either of the RFs of $G_{obj}^{2}$ and $G_{obj}^{3}$ and attention is away from both. In a previous model study, Mihalas *et al*. [26] have argued that attention to an object suppresses activity of nearby *G* cells (*via* IB cells in their model, see their Fig 1). Therefore, when attention is on the center object, bottom row in Table 1, the activity of $G_{obj}^{2}$ and $G_{obj}^{3}$ is set to a low value, half of what it would be without a nearby attended object. Psychophysical and fMRI human imaging studies have reported such inhibitory surround of orientation perception and V1 responses for nearby locations, which are due to the influence of selective attention [48, 49].

Finally, column ‘*G*_*sp*_’ of Table 1 shows the firing rates of the second type of *G* cell, which represents the effect of spatial attention, see Fig 2. If attention is on the center region of the scene, as shown in that figure, this cell fires with a frequency of 15Hz (row ‘Bound-attended’), if this region is not attended, its firing rate is 3Hz (rows ‘Unbound-ignored’ and ‘Bound-ignored’).

In the following sections, we report numerical results of simulations of the model defined above. Population results are given for two measures. One is the firing rate of subsets of BOS *neurons*, the other are correlations (loose and tight, see below) between sets of *pairs of neurons*. For the latter, we already have defined consistent and inconsistent pairs of neurons, see Fig 1. For the former, it is important to note that firing rates are a property of neurons, not pairs of neurons. We differentiate between those neurons whose border ownership preference is towards the object centered between their RFs (gray keystone-like object in Fig 1 and gray parallelogram in Fig 2) and those whose border ownership preference is another object. We call the former “preferred neurons” and the latter “non-preferred” neurons. Note that both members of a consistent pair are preferred neurons. At least one of the members of an inconsistent pair is a non-preferred neuron, the other can be preferred or non-preferred, see Fig 1.

### Mean firing rates of BOS neurons

**Fig 3:**
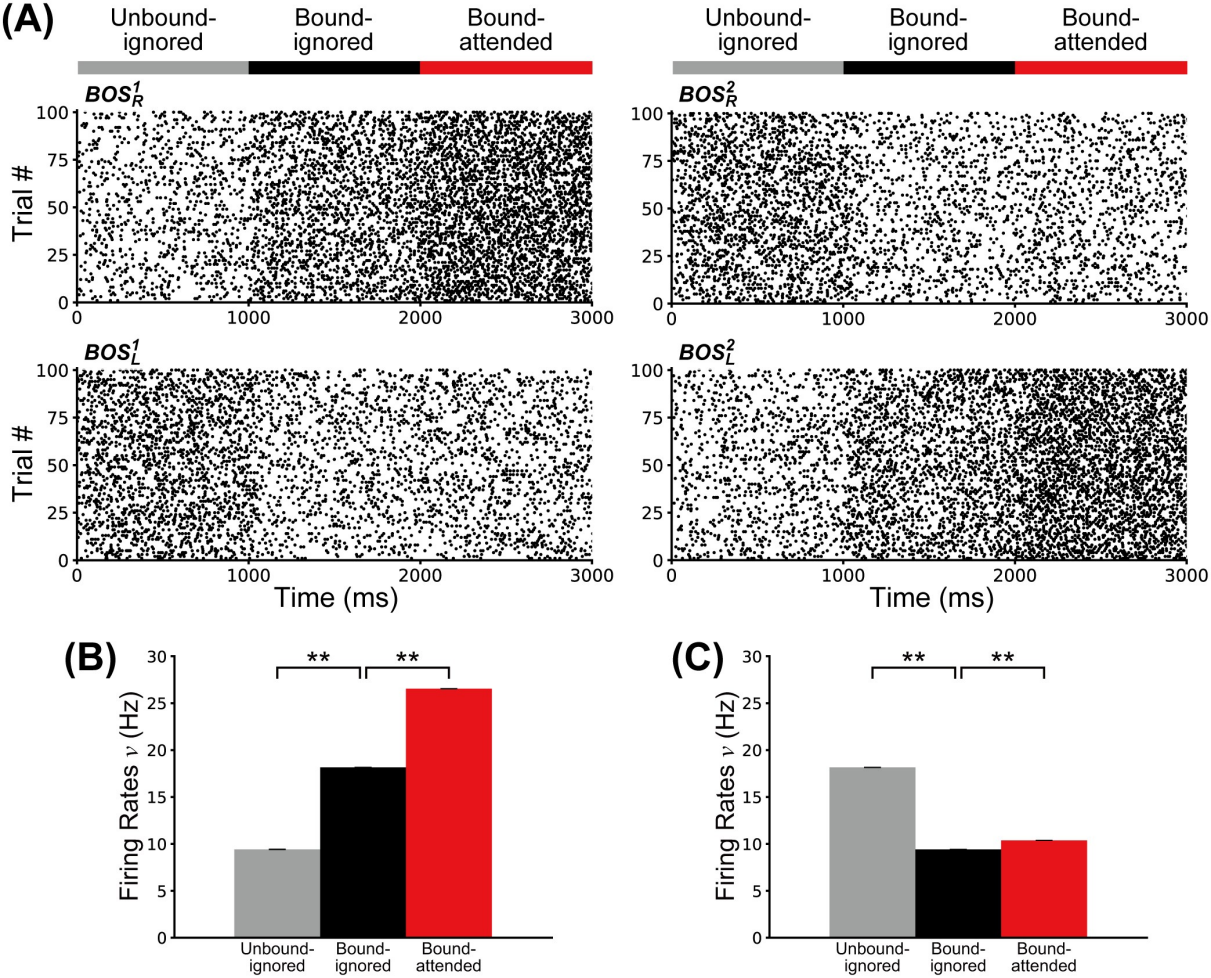
Responses of BOS neurons. A: Raster plots showing 100 spike trains of BOS model neurons. For these plots, G-cells were activated for representing the Unbound-ignored condition between 0 and 1000 ms, the Bound-ignored condition for 1000 and 2000 ms, and the Bound-attended condition for 2000 and 3000 ms. Feedback from G-cells modulates the firing rates of BOS neurons. Identities of BOS neurons are shown next to each plot. B: Firing rates of preferred neurons ($BOS_{R}^{1}$ and $BOS_{L}^{2}$). C: Firing rates of non-preferred neurons ($BOS_{L}^{1}$ and $BOS_{R}^{2}$). The gray, black, and red bars represent the Unbound-ignored, Bound-ignored, and Bound-attended conditions, respectively, during trials that mimic the experimental settings of the Martin and von der Heydt study (ref. [39], their Fig 4B). These firing rates were obtained from 10 sets of 100 simulated trials, each of a length of 200 biological seconds. Error bars indicate standard deviations (SDs), which were very small in these simulations. Confidence intervals of preferred neurons were 9.42 ± 0.01 (*SD* = 0.02), 18.15 ± 0.01 (*SD* = 0.02), and 26.54 ± 0.01 (*SD* = 0.02) Hz for the Unbound-ignored, Bound-ignored, and Bound-attended conditions, respectively. Confidence intervals of non-preferred neurons were 18.15 ± 0.01 (*SD* = 0.02), 9.41 ± 0.01 (*SD* = 0.01), and 10.38 ± 0.01 (*SD* = 0.01) Hz for the Unbound-ignored, Bound-ignored, and Bound-attended conditions, respectively. Asterisks indicate significant differences between conditions (** *p* < 0.01 by t-test).

First, we investigate the influence of *G*-cell activity levels on the spiking frequencies of model BOS neurons. Fig 3A shows spike raster plots of BOS neurons for 100 simulated trials where G-cells are activated in the Unbound-ignored condition between 0 and 1000 ms, in the Bound-ignored condition between 1000 and 2000 ms, and in the Bound-attended condition otherwise. The feedback from G-cells modulates the activities of all BOS neurons.

Fig 3B and 3C show the mean firing rates of BOS neurons for the Unbound-ignored (gray), Bound-ignored (black) and Bound-attended (red) conditions (see Fig 1 for definitions of these terms), in the preferred (Fig 3B) and non-preferred conditions (Fig 3C). Note that the firing rates of preferred neurons are significantly higher in the bound condition than in the unbound condition (Fig 3B; t-test, *p* = 4.9 × 10^−44^, effect size *r* = 1.0). They are also significantly higher in the Bound-attended condition than in the Bound-ignored condition (t-test, *p* = 3.6 × 10^−44^, *r* = 1.0), which is in agreement with physiological results [17, 39]. In contrast to the preferred neurons, the firing rates of the non-preferred neurons are significantly higher in the unbound condition than in the bound condition (Fig 3C; t-test, *p* = 2.7 × 10^−45^, *r* = 1.0). The firing rates of these model neurons are also slightly but significantly increased in the Bound-attended condition relative to the Bound-ignored condition (t-test, *p* = 2.1 × 10^−30^, *r* = 1.0). These results imply that the activities of *G*-cells significantly modulate the spike frequency of BOS neurons in our proposed network.

### Loose synchrony of BOS neurons

We next quantify the loose (correlations on the order of tens of milliseconds) and tight (order of milliseconds) synchrony between BOS neurons of consistent and inconsistent pairs (Fig 1B) using the methods of ref [41].

**Fig 4:**
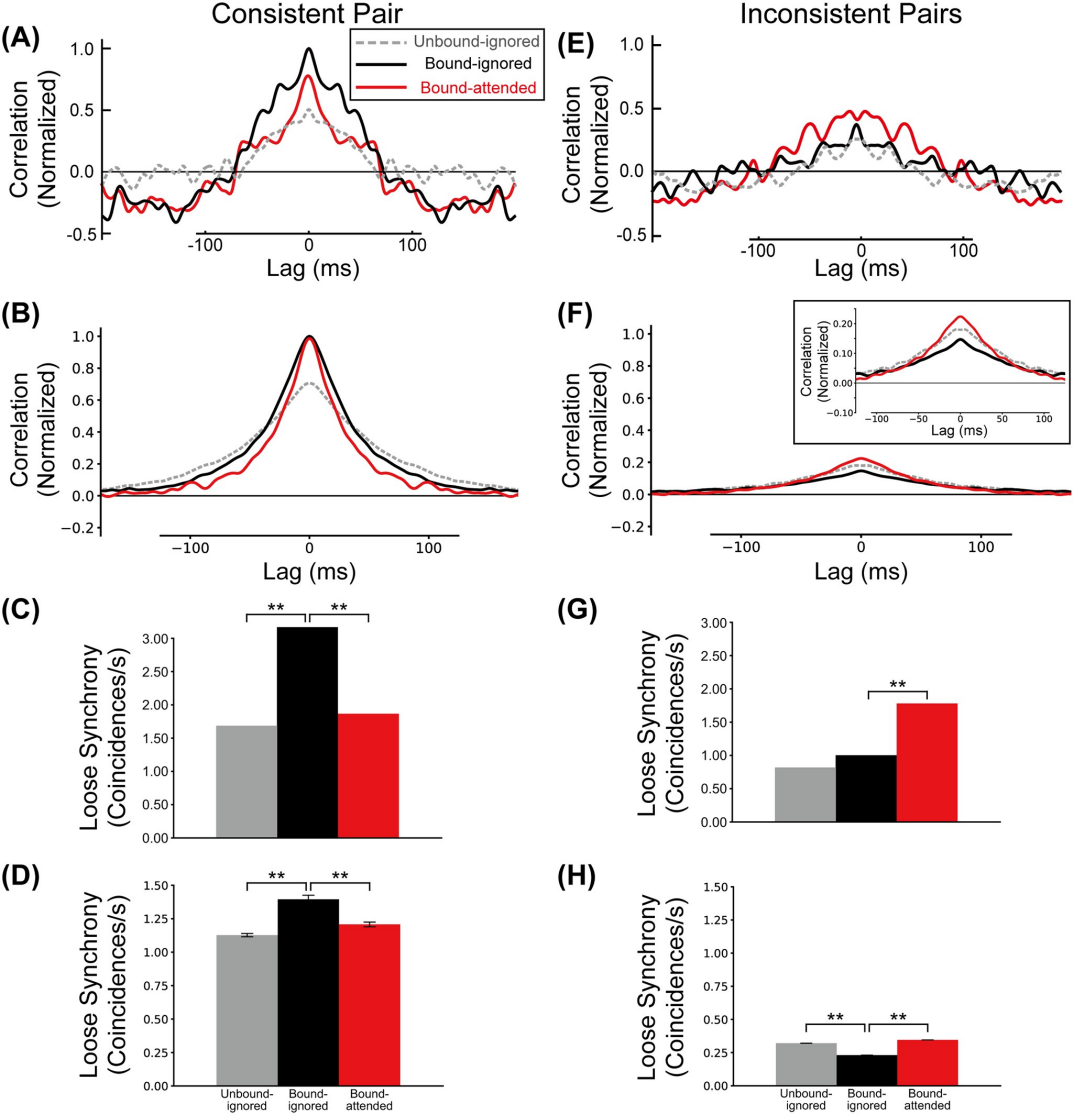
Correlations between BOS neurons. The gray, black, and red lines/bars represent the correlation (loose synchrony) of the Unbound-ignored, Bound-ignored, and Bound-attended conditions, respectively. A, C: Experimentally observed mean spike train cross-correlation and loose synchrony for consistent BOS neurons, modified from [39]. B, D: Model spike train cross-correlation and loose synchrony for the consistent pair. Confidence intervals of loose synchrony of this pair for the Unbound-ignored, Bound-ignored, and Bound-attended (panel D) were 1.13 ± 0.01 (*SD* = 0.01), 1.39 ± 0.02 (*SD* = 0.03), and 1.21 ± 0.01 (*SD* = 0.02) *coincidences*/*s*, respectively. E, G: Experimentally observed mean spike train cross-correlation and loose synchrony for inconsistent BOS neurons. F, H: Model spike train cross-correlation and loose synchrony for the inconsistent pairs. Inset in F shows detail of center region at higher scale. Confidence intervals of loose synchrony of these pairs for the Unbound-ignored, Bound-ignored, and Bound-attended (panel H) were 0.30 ± 0.01 (*SD* = 0.02), 0.23 ± 0.01 (*SD* = 0.01), and 0.34 ± 0.01 (*SD* = 0.01) *coincidences*/*s*, respectively. Curves in all panels are normalized by the maximum correlation value of the consistent pair. The observed maximum values were: A:54, B:24, E: 26, F:5 *coincidences*/*s*^2^. Asterisks indicate significant differences between conditions (** *p* < 0.01, t-test). Error bars indicate SDs.

We define loose synchrony as the integral of spike correlations in the range of a ±40ms interval around lag zero (see Materials and methods section). Loose correlations between $BOS_{R}^{1}$ and $BOS_{L}^{2}$ neurons (the consistent pair) with respect to the Unbound-ignored, Bound-ignored and Bound-attended conditions are shown in Fig 4B. In the ignored conditions (Unbound-ignored and Bound-ignored), the correlation for the bound configuration (black line in Fig 4B) is markedly stronger than in the unbound configuration (gray line in Fig 4B). In contrast, the correlation for the Bound-attended condition (red line in Fig 4B) is weaker than that for the Bound-ignored condition. These bound-induced increase and attention-induced decrease of spike correlations are in qualitative agreement with the experimentally observed response modulation for consistent pairs of BOS neurons [39]. Loose synchrony for the consistent pair in our simulations is summarized in Fig 4D. We found a significant increase in loose synchrony from the Unbound-ignored condition (gray bar in Fig 4D) compared to the Bound-ignored condition (black bar) (t-test, *p* = 4.2 × 10^−15^, *r* = 0.98). In contrast, the loose synchrony significantly decreases from the Bound-ignored condition to the Bound-attended condition (red bar in Fig 4D) (t-test, *p* = 5.7 × 10^−12^, *r* = 0.97). These simulated results agree with those found for consistent BOS neurons [39], Fig 4A and 4C.

Martin and von der Heydt [39] also reported synchrony results for inconsistent pairs (Fig 4E and 4G). These pairs were defined as all possible pairs of BOS neurons with the exception of the consistent pair, as illustrated in Fig 1B. In our model, the inconsistent pairs are represented by three pairs; $BOS_{R}^{1}-BOS_{R}^{2}$, $BOS_{L}^{1}-BOS_{L}^{2}$, and $BOS_{L}^{1}-BOS_{R}^{2}$. The spike-spike correlation of the inconsistent pairs is calculated using the mean of the correlations for these three pairs (Fig 4F). In contrast to the consistent pair (Fig 4B), the correlation for the Bound-attended condition (red line in Fig 4F) exceeds that for the ignored conditions (gray and black lines for the Unbound-ignored and Bound-ignored conditions in Fig 4F, respectively). This is consistent with the experimentally observed modulation pattern of inconsistent pairs, Fig 4E.

We also computed loose synchrony of the inconsistent pairs by integrating the correlation in the range of ±40ms interval around lag zero (Fig 4H). Conventions are the same as those in Fig 4D. The level of loose synchrony for the Bound-attended condition (red bar in Fig 4H) is significantly higher than that for the Bound-ignored condition (black bar in Fig 4H)(t-test, *p* = 2.8 × 10^−16^, *r* = 0.99). Even though loose synchrony for the Unbound-ignored condition (gray bar in Fig 4H) is at a similar level to that for the Bound-attended condition, technically it is significantly different (t-test, *p* = 3.9 × 10^−6^, *r* = 0.84) because of the large amount of simulated data (10 sets of 100 simulated trials). In contrast to the consistent pair (Fig 4D), we found that the loose synchrony for the Bound-ignored condition is the lowest among these three conditions. These modulation patterns of loose synchrony for the inconsistent pairs are opposite to those for the consistent pair. This attentional enhancement of loose synchrony for the inconsistent pairs agree with that of experimentally observed BOS neurons [39]. Our simulation results show that the interaction between two distinct types of attentional feedback signals can explain why correlations between consistent and inconsistent pairs of BOS neurons are of opposite polarity. We investigate the mechanisms underlying this surprising result in sections below.

To further quantify the responses of our model, we compute the noise correlation between BOS neurons of consistent and inconsistent pairs (S1 Fig). The noise correlation for the consistent pair with respect to the Unbound-ignored, Bound-ignored and Bound-attended conditions are summarized in S1(A) Fig. There is no significant difference in the noise correlation between ignored conditions (Unbound-ignored and Bound-ignored) (t-test, *p* = 0.97, *r* = 0.01). By contrast, we found a significant decrease in noise correlation between the Bound-ignored condition and the Bound-attended condition (t-test, *p* = 9.4 × 10^−3^, *r* = 0.57). S1(B) Fig shows the noise correlation of the inconsistent pairs. As for the consistent pairs, we found no significant difference between ignored conditions (Unbound-ignored and Bound-ignored) (t-test, *p* = 0.35, *r* = 0.22). Different from the consistent pair, there is also no significant difference between the noise correlation in Bound-attended and the Bound-ignored conditions (t-test, *p* = 0.48, *r* = 0.17).

One plausible cause of synchrony between spike trains of two BOS neurons is common input to both. Correlation functions of the inputs to BOS neurons are shown in S2 Fig. There are no significant differences in the means of the loose synchrony between *G*_*sp*_- and $G_{obj}^{1}$-cells between Bound-ignored and Bound attended conditions (t-test, *p* = 0.97, *r* = 0.01) nor between Unbound-ignored and Bound-ignored conditions (t-test, *p* = 0.092, *r* = 0.39), S2(B) Fig. Due to the higher firing rate of the G-cell in the Bound-attended cognition, the variance of the correlation function is, however, higher than in the ignored conditions. We also compute spike correlations between bottom-up visual inputs for consistent (S2(C) Fig) and inconsistent pairs (S2(E) Fig). There are no significant differences in levels of loose synchrony in bottom-up visual inputs for the consistent pair (S2(D) Fig; t-test for Unbound-ignored *vs*. Bound-ignored, *p* = 0.43, *r* = 0.19; t-test for Bound-ignored vs. Bound-attended, *p* = 0.67, *r* = 0.22). For inconsistent pairs, no significant differences between the levels of loose synchrony of bottom-up visual inputs were observed (t-test for Unbound-ignored *vs*. Bound-ignored, *p* = 0.51, *r* = 0.15; t-test for Bound-ignored *vs*. Bound-attended, *p* = 0.35, *r* = 0.22) (S2(F) Fig). Of course, this lack of significant correlation is entirely expected because in our simulation, the input consists of independent Poisson processes. For this reason, despite the lack of significant differences in the means of cross-correlation functions between G-cells, we surmise that correlations between BOS neurons are induced by the common feedback from *G*-cells to BOS neurons.

The neurophysiological data in Fig 4A and 4E show some evidence of periodicity in the beta/gamma range which is absent in the simulated data. Martin and von der Heydt [39] did not found statistically significant differences in this spectral range between the different experimental conditions (see their discussion of their Fig 4 on which our Fig 4 is based). This was the case for both spike-spike and spike-field coherence. We therefore do not assign significance to these oscillations.

### Tight synchrony of BOS neurons

Martin and von der Heydt [39] investigated tight synchrony [50, 51] between BOS neurons with respect to consistent and inconsistent pairs using a transformation of the original spike trains in which spikes are distributed randomly within a jitter window of width Δ = 20*ms* (jittered spike train). A large number of correlations between jittered spike trains were computed, and their mean was subtracted from the original spike correlation, resulting in the jitter-reduced correlation (tight correlation). This procedure removes all correlations at times scales larger than the jitter window Δ, revealing the underlying tight synchrony [50, 51]. A detailed description of the procedure for computing tight correlation and synchrony is given in the Materials and Methods section. We computed tight synchrony by integrating the tight correlation in the range of ±5ms interval around lag zero.

**Fig 5:**
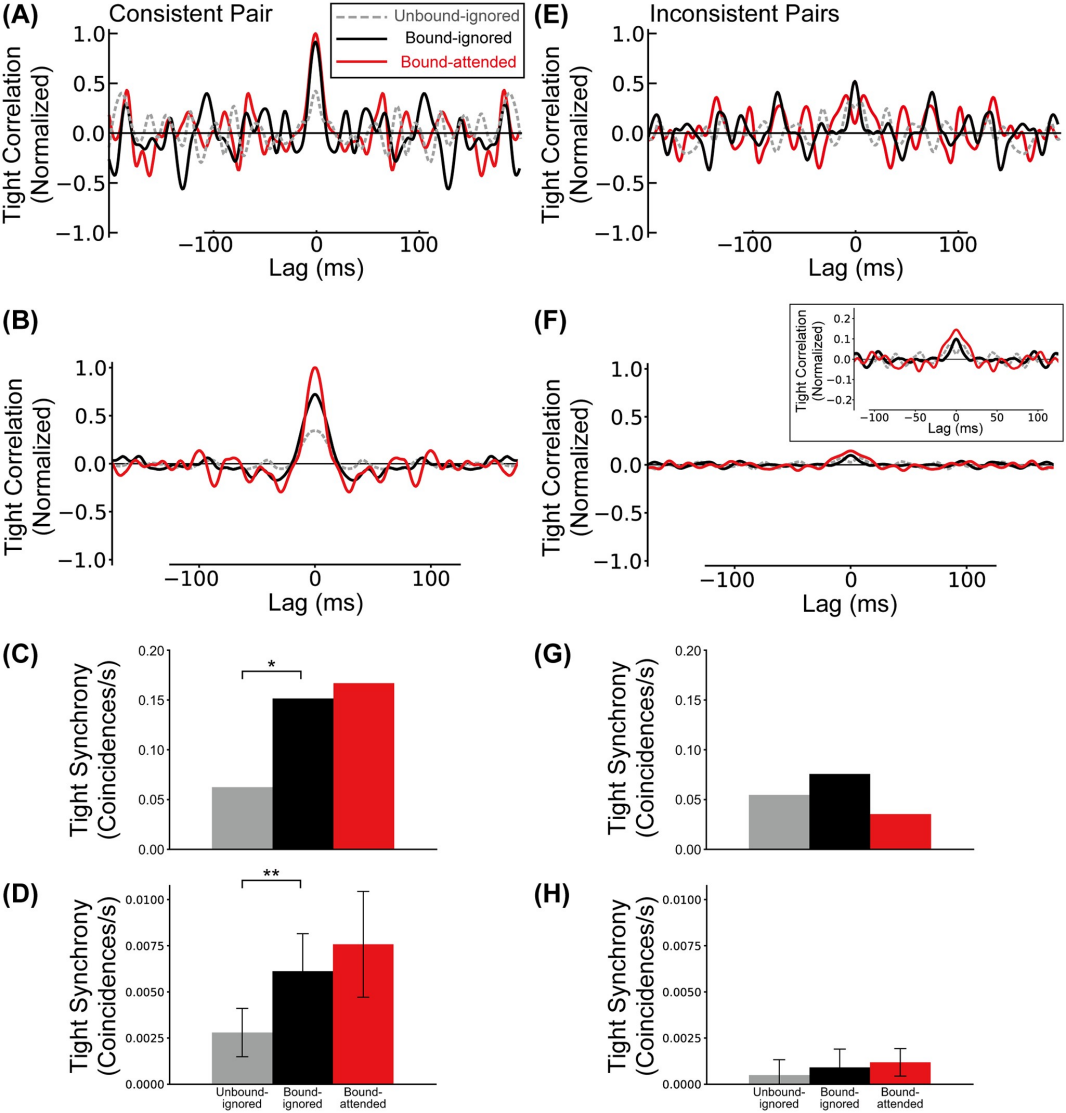
Tight synchrony between model BOS neurons. Reduced cross-correlations after subtraction of Δ = 20*ms* interval jitter cross-correlation. A, C: Experimentally observed mean tight correlation and tight synchrony of the consistent pair for the Unbound-ignored (gray), Bound-ignored (black), and Bound-attended (red) conditions from [39]. B, D: Simulated tight correlation and tight synchrony of the consistent pair. Conventions are the same as those in A and C. Confidence intervals of tight synchrony of this pair for the Unbound-ignored, Bound-ignored, and Bound-attended conditions (panel D) were 0.0028 ± 0.0008 (*SD* = 0.0013), 0.0061 ± 0.0013 (*SD* = 0.0020), and 0.0076 ± 0.0018 (*SD* = 0.0029) *coincidences*/*s*, respectively. E, G: Experimentally observed mean tight correlation and tight synchrony of the inconsistent pair. F, H: Simulated tight correlation and tight synchrony of the inconsistent pairs. Inset in the panel F shows central area at higher scale. Confidence intervals of tight synchrony of these pairs for the Unbound-ignored, Bound-ignored, and Bound-attended conditions (panel H) were 0.0005 ± 0.0005 (*SD* = 0.0008), 0.0009 ± 0.0006 (*SD* = 0.0010), and 0.0012 ± 0.0005 (*SD* = 0.0007) *coincidences*/*s*, respectively. Curves in all panels are normalized by the maximum value of the “Bound-attended” condition of the consistent pair. The observed maximal values were: A:17.4, B:3.7, C:3.4, D:0.5 *coincidences*/*s*^2^. Asterisks indicate significant differences between conditions (* *p* < 0.05, ** *p* < 0.01, t-test). Error bars indicate SDs.

The experimentally observed tight correlations for the consistent pair shows significant peaks at zero lag in the bound conditions, but not in the unbound condition [39]. Data are reproduced in Fig 5A which shows the tight correlations of the consistent pair for three conditions. Under the bound conditions (black and red lines in Fig 5A), there are marked peaks of tight correlation around zero lag. Fig 5B shows the corresponding curves from our simulation of the model, obtained by applying the same jitter method as in the experimental results to our simulation data. Tight synchrony for the consistent pair in physiological experiments and our simulations are summarized in Fig 5C and 5D, respectively. Fig 5E shows experimental tight correlation for the inconsistent pairs and Fig 5F the corresponding simulation results. Experimentally observed tight synchrony for inconsistent pairs is shown in Fig 5G. Fig 5H summarizes model tight synchrony for inconsistent pairs.

Irrespective of grouping structure and attention conditions, inconsistent pairs in the experiment did not show a significant peak of tight correlation at zero lag [39](Fig 5E). We computed tight correlation based on our simulation data for inconsistent pairs (Fig 5F). There was no marked peak around zero lag under Unbound-ignored condition (gray line). By contrast, under the bound conditions, we found peaks of tight correlation for inconsistent pairs (black and red lines). However, the peak value of tight correlation for the inconsistent pairs under the Bound-attended conditions (see caption of Fig 5) was markedly weaker than that for the consistent pair. Overall, tight correlation for inconsistent pairs in our model is similar to that observed physiologically. No significant differences between the levels of tight synchrony of inconsistent pairs was observed in Martin and von der Heydt’s electrophysiological recordings [39] (Fig 5H). They found, however, that in the Bound-ignored condition, tight synchrony between members of consistent pairs was significantly higher than that of inconsistent pairs (Fig 5C and 5G). Likewise, simulation of the Bound-ignored condition showed a significantly higher tight synchrony between consistent pairs (the black bar in Fig 5D) than between inconsistent pairs (the black bar in Fig 5H) (t-test, *p* = 1.8 × 10^−6^, *r* = 0.85). Additionally, there were no significant differences between levels of tight synchrony of inconsistent pairs in our simulation results (Fig 5H; t-test for Unbound-ignored *vs*. Bound-ignored, *p* = 0.35, *r* = 0.22; t-test for Bound-ignored vs. Bound-attended, *p* = 0.52, *r* = 0.15), which were similar characteristics to electrophysiological results [39]. Note the different observed maximal values for each subplot, which is listed in the caption of Fig 5. We next explore the differential role of the two types of grouping cells in our model and their contributions to the observed loose and tight synchrony results.

### Modulation of BOS cell firing rates by top-down input

Results so far have characterised activity levels (firing rates) and pairwise correlations of BOS neurons for a fixed set of firing rates of the *G* cells that were either chosen according to previous firing rate models ($G_{obj}^{1}$-cells) or just assumed (*G*_*sp*_-cell). To understand the circuit behavior, we now systematically vary those *G*-cell firing rates and report the effect on the firing rates (this section) and correlations (following two sections) of BOS neurons.

**Fig 6:**
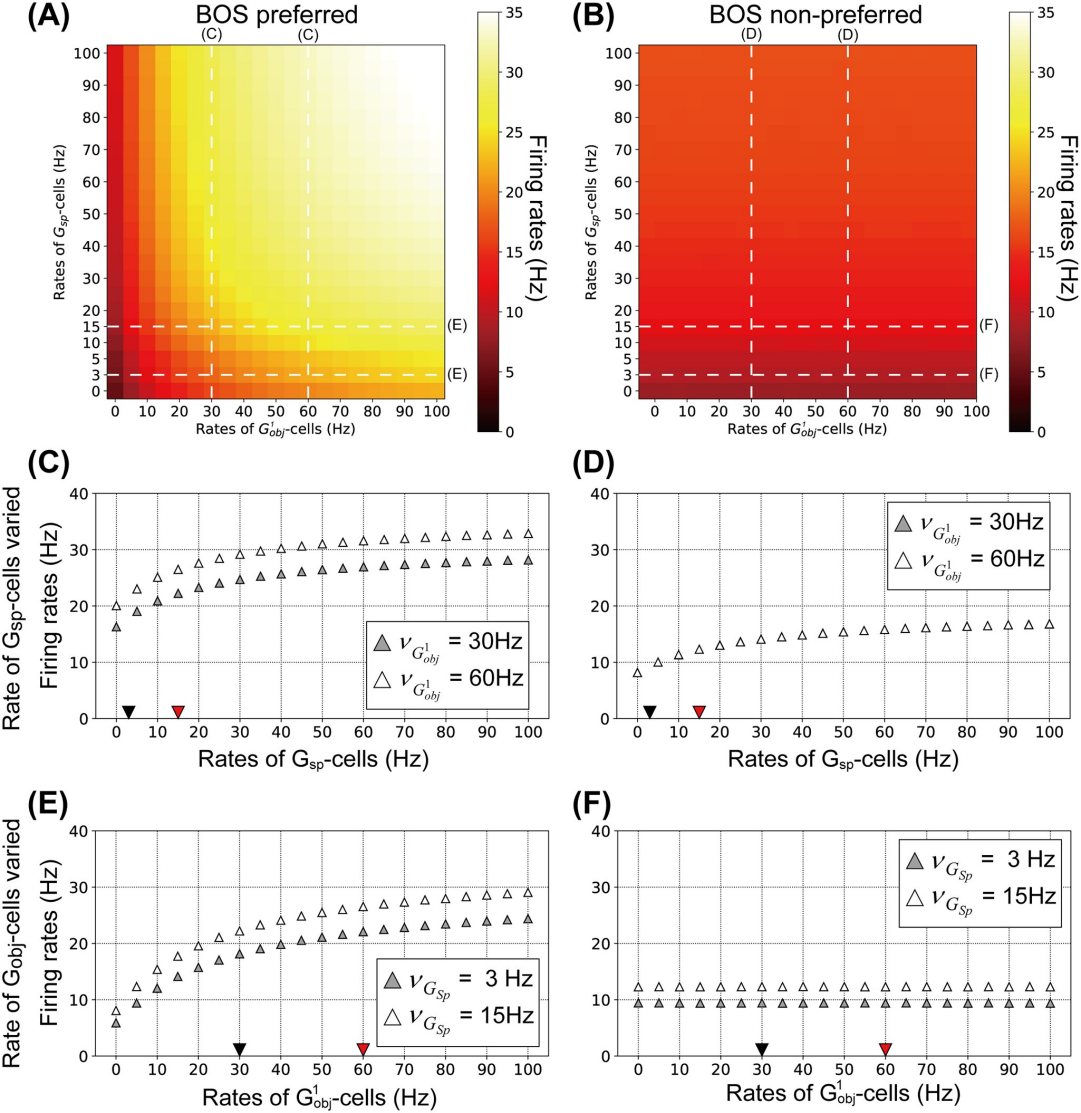
Firing rates for BOS neurons as functions of the mean rates of *G*-cells. A: Averaged firing rates of $BOS_{L}^{1}$ and $BOS_{R}^{2}$ neurons for combinations of $G_{obj}^{1}$- and *G*_*sp*_-cells rates. B: Averaged firing rates of $BOS_{R}^{1}$ and $BOS_{L}^{2}$ neurons for combinations of $G_{obj}^{1}$- and *G*_*sp*_-cells rates. Firing rates of BOS neurons pointed out by white dashed lines with ‘C’, ‘D’, ‘E’ and ‘F’ are summarized in panels C, D, E and F as a functions of the mean rates of *G*-cells, respectively. In C-F, each data point is the average of 100 simulated trials, each 200 biological seconds long as a function of the mean rate of *G*-cells. C, D: Firing rates of BOS neuron as function of the mean rates of *G*_*sp*_ for two mean rates of $G_{obj}^{1}$, 30Hz (filled triangles) and 60Hz (open triangles). C: Average of $BOS_{R}^{1}$ and $BOS_{L}^{2}$. Black and red triangles indicate the chosen *G*_*sp*_ rates for ignored and attended conditions, respectively. D: Average of $BOS_{L}^{1}$ and $BOS_{R}^{2}$ neurons. Note that symbols for 30Hz and 60Hz nearly overlap because there is no direct projection from object-based G-cells to members of the inconsistent BOS cell pairs. E, F: Firing rates of BOS neurons as functions of the mean rates of $G_{obj}^{1}$ for two different mean rates of *G*_*sp*_, 3Hz (filled triangles) and 15Hz (open triangles). E: Average of $BOS_{R}^{1}$ and $BOS_{L}^{2}$. Black and red triangles indicate the chosen rates of $G_{obj}^{1}$-cells for bound-ignored and bound-attended conditions, respectively. F: Average of $BOS_{L}^{1}$ and $BOS_{R}^{2}$.

Firing rates of BOS neurons for a variety of combinations of $G_{obj}^{1}$- and *G*_*sp*_-cell rates for consistent and inconsistent pairs are summarized in Fig 6A and 6B, respectively. The preferred $BOS_{R}^{1}$ and $BOS_{L}^{2}$ neurons are activated with increasing the rates of both $G_{obj}^{1}$- and *G*_*sp*_-cells (Fig 6A). Rates of the non-preferred $BOS_{L}^{1}$ and $BOS_{R}^{2}$ neurons are also moderately increased with increasing the rates of *G*_*sp*_-cells (Fig 6B). However, the activation of $G_{obj}^{1}$-cells does not modulate the responses of non-preferred BOS neurons.

Fig 6C shows the firing rates of the preferred $BOS_{R}^{1}$ and $BOS_{L}^{2}$ neurons and Fig 6D that of the non-preferred $BOS_{L}^{1}$ and $BOS_{R}^{2}$ neurons as a function of the rates of the *G*_*sp*_-cell. In these cases, $G_{obj}^{1}$-cells fire with a mean frequency of 30Hz (filled triangles) or 60Hz (open triangles). The firing rates of all BOS neurons increase monotonically with increasing activity of the *G*_*sp*_-cell. Note that in panel D, the symbols overlap because the non-preferred neurons $BOS_{L}^{1}$ and $BOS_{R}^{2}$ do not receive direct input from $G_{obj}^{1}$ (see Fig 2) and therefore there is no noticeable difference between the 30Hz and 60Hz curves.

Fig 6E shows the mean rates of preferred BOS neurons as a function of the firing rate of $G_{obj}^{1}$ for *G*_*sp*_-firing rates of 3Hz (open triangles) and 15Hz (filled triangles). It is seen that rates increase monotonically. The analogous result for non-preferred neurons is shown in panel F of that figure. Their rates are independent of the activity of $G_{obj}^{1}$, since this neuron does not project directly to $BOS_{L}^{1}$ and $BOS_{R}^{2}$, see Fig 2.

### Modulation of loose synchrony between BOS cells by top-down input

**Fig 7:**
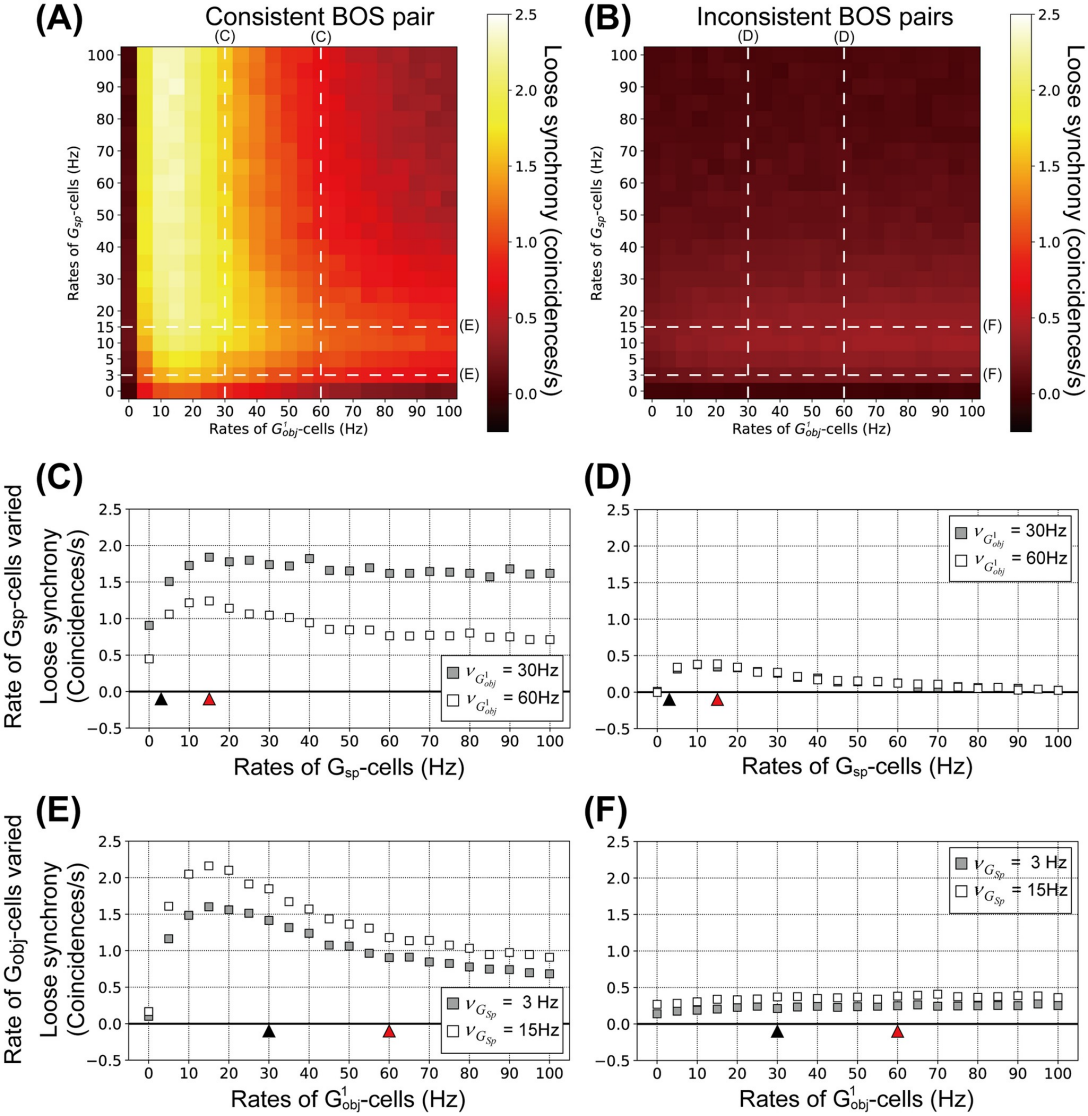
Loose synchrony for model BOS neurons as function of the mean rates of *G*-cells. Shown is the integral of the spike train cross-correlation in the range of a ±40 ms interval around lag zero. Each data point is the average of 100 simulated trials, each of 200 biological seconds duration. A: Loose synchrony between $BOS_{R}^{1}$ and $BOS_{L}^{2}$ neurons (consistent pair) as a function of combinations of $G_{obj}^{1}$- and *G*_*sp*_-cells rates. B: Loose synchrony between all inconsistent pairs (defined in Fig 1B) of BOS neurons as function of combinations of $G_{obj}^{1}$- and *G*_*sp*_-cells rates. Modulation patterns of loose synchrony labelled by white dashed lines with ‘C’, ‘D’, ‘E’ and ‘F’ are summarized in panels C, D, E and F, respectively. C: Loose synchrony between consistent pair as a function of the mean rate of *G*_*sp*_ for two different mean rates of $G_{obj}^{1}$, 30Hz (filled squares) and 60Hz (open squares). Black and red triangles are the same as those in Fig 6C. D: Loose synchrony between all inconsistent pairs (defined in Fig 1B) of BOS neurons as function of the rates of *G*_*sp*_. Note that symbols of $G_{obj}^{1}$ for 30Hz and 60Hz nearly overlap because there is no direct projection from $G_{obj}^{1}$-cells to members of the inconsistent BOS cell pairs. Conventions are the same as in panel C. E: Loose synchrony between consistent pair as function of the mean rates of $G_{obj}^{1}$ for different mean rates of *G*_*sp*_, of 3Hz (filled squares and 15Hz (open squares). Black and red triangles are the same as those in Fig 6E. F: Loose synchrony between inconsistent pairs of BOS neurons as function of the rates of $G_{obj}^{1}$. Conventions as in panel E.

The influence of *G*-cell firing rates on loose synchrony between BOS cells is summarized in Fig 7. Fig 7A and 7B shows loose synchrony of BOS neurons with various combinations of $G_{obj}^{1}$- and *G*_*sp*_-cell rates for consistent and inconsistent pairs, respectively. Loose synchrony for the consistent pair is increased for firing rates of $G_{obj}^{1}$-cells in the range 10∼20 Hz (Fig 7A). By contrast, higher activation of both $G_{obj}^{1}$- and *G*_*sp*_-cells decreases loose synchrony of the consistent pair. For inconsistent pairs, modulations of loose synchrony might be independent of the activation of $G_{obj}^{1}$-cells (Fig 7B). However, activation of *G*_*sp*_-cells modulates the loose synchrony for both consistent and inconsistent pairs.

Details of modulation in loose synchrony of the consistent and inconsistent pairs as a function of the *G*_*sp*_-cell activity are shown in Fig 7C and 7D, respectively. As in the previous section, in these simulations mean rates of $G_{obj}^{1}$-cells are set to 30Hz or 60Hz. Again, symbols overlap in panel D because rates of neurons forming inconsistent pairs do not receive direct input from $G_{obj}^{1}$.

Loose synchrony for the consistent and inconsistent pairs is a non-monotonic function of $\nu_{G_{sp}}$, rising to a peak at $\nu_{G_{sp}}\approx 15\text{Hz}$ and then decreasing. In addition, for the consistent pair, loose synchrony for $\nu_{G_{obj}^{1}}=60\text{Hz}$ (open squares in Fig 7C) is consistently weaker than for $\nu_{G_{obj}^{1}}=30\text{Hz}$ (filled squares). Fig 7E and 7F show loose synchrony for the consistent and inconsistent pairs as a function of the mean firing rate of $G_{obj}^{1}$, respectively, with *G*_*sp*_ frequency set to 3Hz (filled squares) or 15Hz (open squares). Loose synchrony of the consistent pair as a function of $G_{obj}^{1}$-cells, panel E, show a similar pattern as when *G*_*sp*_ is varied, panel C. In contrast, irrespective of the rate of $G_{obj}^{1}$, loose synchrony for the inconsistent pairs is almost constant, Fig 7F.

### Modulation of tight synchrony between BOS cells by top-down input

**Fig 8:**
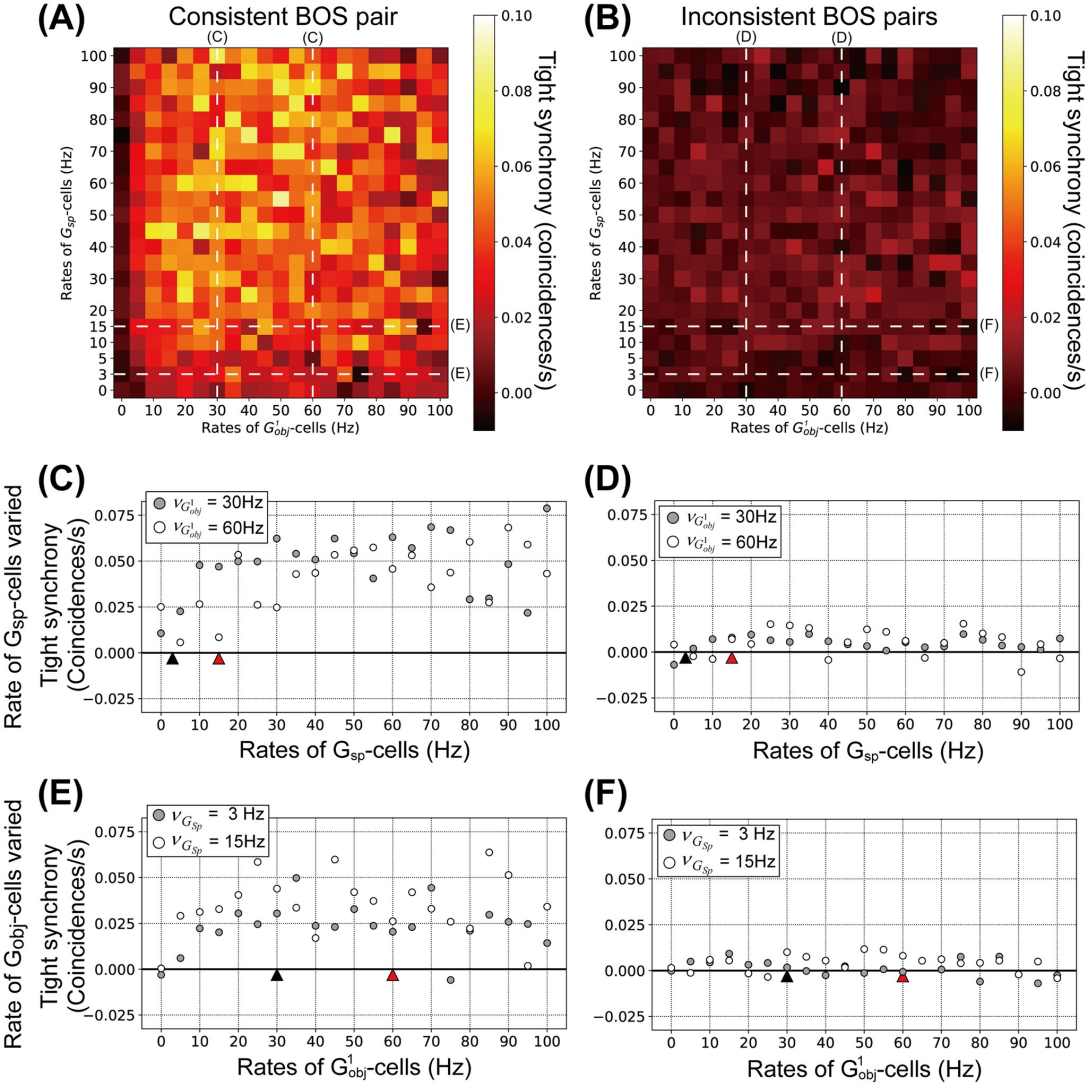
Tight synchrony for model BOS neurons as function of the mean rates of *G*-cells. Shown is the integral of the tight correlation in the range of ±5 ms interval around lag zero. A: Tight synchrony between $BOS_{R}^{1}$ and $BOS_{L}^{2}$ neurons (consistent pair) with respect to combinations of $G_{obj}^{1}$- and *G*_*sp*_-cells rates. B: Tight synchrony between all inconsistent pairs. Modulation patterns of tight synchrony labelled by white dashed lines with ‘C’, ‘D’, ‘E’ and ‘F’ are summarized in panels C, D, E and F as functions of the mean rates of *G*-cells, respectively. C: Tight synchrony for consistent pair as a function of the mean rates of *G*_*sp*_ for mean rates of $G_{obj}^{1}$ of 30Hz (filled circles) and 60Hz (open circles). Black and red triangles are the same as those in Fig 6C. D: Tight synchrony between BOS neurons for inconsistent pairs as a function of the *G*_*sp*_ rate. Conventions as in panel C. E: Tight synchrony between consistent pair as a function of the mean rates of $G_{obj}^{1}$ for mean rates of *G*_*sp*_-cell of 3Hz (filled circles) and 15Hz (open circles). Black and red triangles are the same as those in Fig 6E. F: Tight synchrony between BOS neurons for inconsistent pairs as a function of $G_{obj}^{1}$ rate. Conventions as in panel F.

Finally, we compute tight synchrony between model BOS neurons by systematically varying the rates of *G*-cells (Fig 8). The magnitude of tight synchrony was computed by integrating the tight correlation in the range of ±5 ms around lag zero (see also Materials and methods section). Tight synchrony of BOS neurons with various combinations of rates of $G_{obj}^{1}$- and *G*_*sp*_-cells for consistent and inconsistent pairs is shown in Fig 8A and 8B, respectively. Tight synchrony for the consistent BOS neuron pair (Fig 8A) is markedly higher than that for inconsistent BOS neuron pairs (Fig 8B). However, in contrast to the cases of firing rates (Fig 6A and 6B) and loose synchrony (Fig 7A and 7B), regardless of consistent and inconsistent BOS neuron pairs, there are no specific modulation patterns in tight synchrony as a function of rates of $G_{obj}^{1}$- and *G*_*sp*_-cells.

Fig 8C and 8D show details of tight synchrony modulation for the consistent and inconsistent pairs as a function of *G*_*sp*_-cell firing rates, respectively. Detailed modulations of tight synchrony for the consistent and inconsistent pairs as a function of $G_{obj}^{1}$-cell firing rates are shown in Fig 8E and 8F, respectively. Irrespective of the frequency of *G*-cells, we find higher tight synchrony for the consistent pairs (Fig 8C and 8E) compared to that for the inconsistent pairs (Fig 8D and 8F). The magnitudes of tight synchrony for the inconsistent pairs are close to zero, irrespective of *G*-cell types and their firing rates. In contrast, for the consistent pairs, activation of *G*-cells generates higher levels of tight synchrony. However, the level of tight synchrony (Fig 8) is much more variable compared to that of the loose synchrony (Fig 7).

## Discussion

Our overall goal is to understand the neuronal circuitry responsible for scene understanding and selective attention in primate visual cortex. In this computational study we extend a previously developed neuronal network model with spiking neurons [41] which is based on the grouping hypothesis [16, 25, 26, 28]. Electrophysiological recordings have elucidated the interaction between perceptual organization implemented by border ownership selective neurons and selective attention [17], and the cited grouping models take this “attention to objects” [7, 52] mechanism into account. There is, however, evidence that there are mechanisms of selective attention that act purely spatially, without reference to visual objects [53–55]. It is therefore necessary to expand grouping models to include such purely spatial mechanisms that operate independently of object features.

Our previous grouping model [41] took into account attentional top-down influences of the first kind described above, *i.e*. attention to objects. In that model, *G* cells only project to those BOS cells that are consistent with an object in the attended position, we call these *G*_*obj*_ cells here. In the present study, we extend this model by adding an implementation of the second type of attentional top-down projection which is purely spatial. This projection, implemented by a separate class of *G* cells (*G*_*sp*_) modulates *all* BOS cells in a spatially defined area. This is seen in Fig 2 which shows that the spatial grouping cell *G*_*sp*_ projects to all BOS cells, irrespective of their RF location (in this area) and their border ownership preferences. What is common to both types of grouping cells is that their feedback is modulatory *via* NMDA receptors [42, 43].

To understand the neuronal circuitry we follow a rich and active tradition of theoretical work [32–34] by analyzing the correlation structure of neuronal spike trains, using first-order (mean firing rate) and second order (spike-spike) correlation functions. In particular, common input plays a critical role for inducing synchronized responses between postsynaptic neurons. We focus on the analysis of the observed spike-spike correlations in (mainly) extra-striate cortex. Specifically, we focus on synchrony observed in consistent and inconsistent pairs of BOS neurons (Fig 1).

Our simulations of the new model reproduce the shapes of the cross-correlation functions observed in a recent neurophysiological study [39], for both consistent and inconsistent pairs of BOS neurons, and for all conditions of feature binding and attention. In addition, we showed that: 1) firing rates of BOS cells increase monotonically with increasing *G*-cell activity (cells of inconsistent pairs only with *G*_*sp*_ cell input because they do not receive *G*_*obj*_ cell input), 2) loose synchrony between BOS cells is a non-monotonic function of *G*-cell activity if they both receive common input from the *G*-cell and is stronger in consistent versus inconsistent BOS pairs, and 3) tight synchrony results were more variable, with a higher magnitude of tight synchrony in consistent versus inconsistent BOS pairs. In the next section, we discuss how the top-down influences in our model generate correlation structures that we describe. Overall, our results support the hypothesis that figure-ground organization and attentive selection are both produced by grouping feedback modulation to the early feature representation levels of the visual cortex and suggest that the modulation is mediated by the NMDA-type receptor.

### Mechanisms of spike synchrony between BOS neurons

Neurophysiological data [39] show that attention to a target stimulus has opposite effects on the level of synchrony (In this section, we use synchrony synonymously with loose synchrony except where specifically noted otherwise.) on two neuronal populations: The spike-spike correlation between two neurons whose border ownership preferences point to a common object (which makes them consistent neurons, see Fig 1B) is lowered when attention is directed toward the object. In contrast, the correlation between pairs of neurons whose border ownership preferences do *not* both point to that object, *i.e*. inconsistent neurons, goes up when that object is attended. Our previous work [41] explained the correlation structure for consistent neurons but did not address responses of inconsistent neurons. In the current study, we include this group of neurons and we also specify in more detail the type of attentional influences in our model. As we have discussed earlier, there are multiple mechanisms of top-down visual attention. We here focus on attention to a spatially defined portion of the visual field, *i.e*. spatial attention, and attention to objects.

A fundamental hypothesis of our approach is that these two types of attentional influences have anatomical implementations in the form of separate types of *G* cells. One type consists of object-based *G* (*G*_*obj*_) cells, similar to those in previous studies [25, 26, 41]. Neurons of this type are responsible for grouping (binding) features together to a coherent object and also serve as a “handle” for attention to such an object. This is to be distinguished from spatial attention where *everything* within a spatially circumscribed area is attended. Our hypothesis is that this is implemented by top-down feedback that activates a separate type of neurons, the spatial *G* (*G*_*sp*_) cells. In our model, both types of *G* cells exert attentional influence on BOS cells by NMDA-type projections [43], Fig 2. Furthermore, we assume that top-down attention to one of the target stimuli (shown in schematic form in Fig 1) engages both types of *G* cells, resulting in the elevated firing rates of both cell classes in the attended *vs*. unattended condition, Table 1.

At the level of BOS cells, our simulations show that top-down attention depresses synchrony for the consistent pair, as observed [39]. Our simulation also reproduces the observed *increased* synchrony of inconsistent cell pairs when the object is attended. This difference in population responses can be understood from our model results shown in Fig 7. It shows how synchrony between members of consistent (Fig 7A, 7C and 7E) and inconsistent (Fig 7B, 7D and 7F) pairs varies with the level of common input from *G*_*sp*_ (Fig 7C and 7D) and *G*_*obj*_ (Fig 7E and 7F) cells. As is expected on theoretical grounds, synchrony is a smoothly varying function with a single peak which can be pronounced (Fig 7E) or weak (Fig 7D), except for the inconsistent pairs in which synchrony does not vary with the *G*_*obj*_-cell firing rates (Fig 7F). Top-down attention is assumed to increase firing rates of both *G* cell types, Table 1, which corresponds to a rightward shift along the curves in Fig 7C, 7D, 7E and 7F. The starting point of this shift differs, however, between the two types of *G* cells. *G*_*obj*_-cells fulfill the dual role of mediating attention to objects and of grouping object features pre-attentively. For the center object in Fig 1, the second of these functions (grouping) requires them to fire at a high rate (30Hz, see Table 1) even without attention. This is already to the right of the peak in synchrony, Fig 7E. Attention further increases (doubles) this rate, *i.e*. shifts it to the right in that figure, resulting in a substantially lower synchrony level. Since only consistent BOS cells receive input from the (center) *G* cell, $G_{obj}^{1}$, this effect applies only to them.

*G*_*sp*_-cells provide input to *all* BOS cells in their projective fields. Since they are not involved in feature binding, their firing rates are much lower than those of *G*_*obj*_-cells. This has the important effect that the rightward shift in Fig 7 occurs *from the left* of the maximum, *i.e*. it increases the synchrony level at the level of BOS cells. This explains the increased synchrony between inconsistent cells because, except for low levels of spontaneous firing that is always present in all *G* cells, their only common top-down input is from *G*_*sp*_-cells. This slight increase is also present in consistent cells but it is masked by the larger decrease resulting from the input from the *G*_*obj*_-cells discussed above.

Martin and von der Heydt [39] also reported that tight synchrony shows peaks at zero lag for the consistent pair in the bound condition, but not for the inconsistent pair (Fig 5A and 5E). Our model reproduces the characteristics of tight synchrony, provided we choose synaptic weights of *G*_*obj*_-cells twice as large as that of *G*_*sp*_-cells (see Materials and methods section), a prediction of our model. The differences in synaptic weights between *G*_*obj*_- and *G*_*sp*_-cells underlie that difference in tight synchrony between consistent and inconsistent pairs.

The relative rates of object-based and spatial *G* cells in Table 1 are thus an important prediction of our model (and more specific than the prediction that these two neuronal populations exist in the first place). In this view, the primary function of the object-based *G* cells is grouping of object features, and this functionality is modulated by attention to objects. In contrast, the spatial *G* cells only have *one* function. This difference in functionalities is reflected in their mean firing rates and leads, indirectly, to the observed difference in correlations at the BOS cell level.

A variety of studies have investigated the role of synchrony between neuron pairs for processing visual information and for organizing visual perception [18, 56, 57]. By contrast, our work suggests the possibility that the observed synchrony between BOS neurons is epiphenomenally induced via modulatory feedback mediated by currents through NMDA receptors. Further studies are necessary for understanding the roles of spike synchrony between pairs of BOS neurons.

### Differentiation of foreground and background objects in cluttered scenes

In our model, the state of object representations (attended *vs*. ignored, bound *vs*. unbound) is represented by the mean firing rates of their associated *G* cells, see Table 1. We do not model in detail the mechanisms that create the differences in *G* cell firing rates since we focus in this study on the effects they have on the activity of border ownership selective cells and their correlations.

As far as the effect of attention is concerned, the assumption (made explicit in Table 1) is that attention to an object doubles the firing rate of its associated object-based *G* cell population (from 30Hz to 60Hz) and it also halves the firing rates of nearby *G* cells of the same type (from 5Hz to 2.5Hz) because of interactions between *G* cells [26]. The increase is assumed to result from top-down input from the attention control system that provides additional excitatory input to the *G* cells representing attended objects.

There is also a strong difference between object-based *G* cells representing bound and unbound regions of the RF, with firing rates assumed as 30Hz in the former and 5Hz in the latter case (in the absence of attention; attention to an object doubles the rate as discussed). The origin of this difference is the different input from BOS cells in the bound *vs*. unbound conditions. As Fig 2 shows, $G_{obj}^{1}$ receives input from a large population of BOS cells on the borders in the bound condition (grey parallelogram) and it stands to reason that this *G* cell has therefore a higher firing rate than the neighboring *G* cells $G_{obj}^{2}$ and $G_{obj}^{3}$ that represent unbound regions with a much smaller number of active BOS cells feeding into them. This explains the large difference between *G* cells representing bound and unbound features.

**Fig 9:**
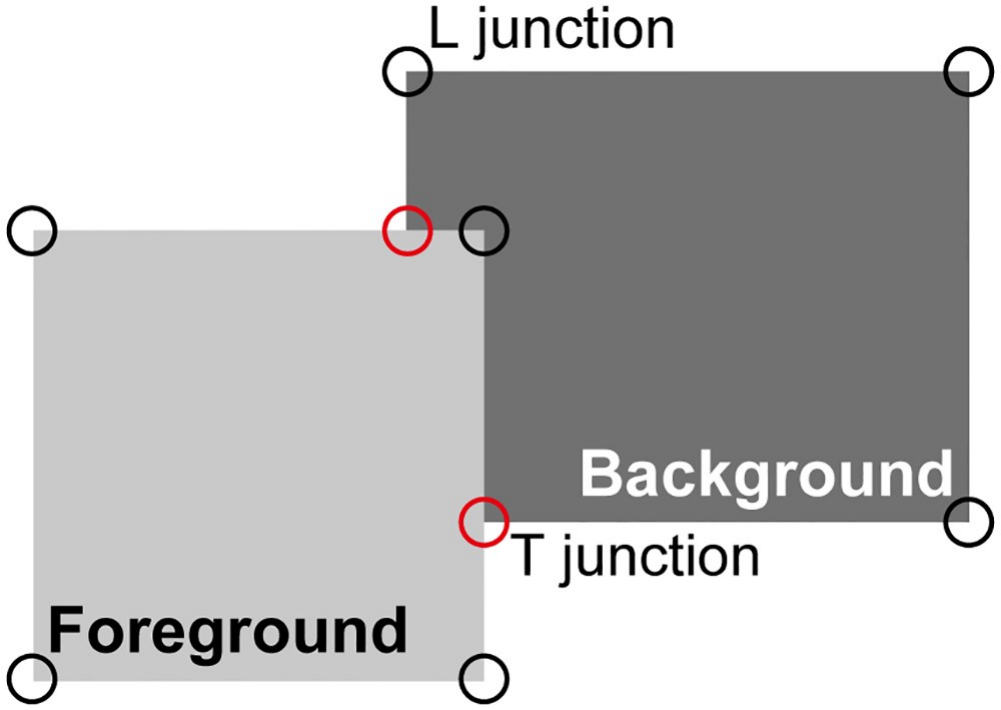
Overlapping squares as an example of cluttered scenes. In this illustration, the light-gray “foreground” square appears in front of the darker “background” rectangle; the perception is not that of a light-gray square adjacent to a darker L-shaped object. Red and black circles mark T and L junctions, respectively, and are not part of the visual display. Such local cues modulate the responses of BOS cells in addition to the grouping cell inputs discussed in the present study [25].

However, one might argue that the latter argument, that firing rates differ substantially between bound and unbound object representations, may hold for simple scenes with isolated objects as in Fig 2 but not necessarily for more complex scenes. In particular, we need to consider the presence of clutter and overlapping objects. An example is the simple scene showing partially overlapping rectangles shown in Fig 9. If the circuitry shown in Fig 2 receives input from this or a similar scene, the object-based *G* cells that receive input from the background figure (dark gray rectangle) will have a similar firing rate as the object-based *G* cell that represents the foreground figure (light gray rectangle) since both receive input from about the same number of line segments. Thus, our assumption that the *G* cell of one of them (the foreground figure) has a substantially higher firing rate seems not justified.

This is certainly the case for the model described so far. However, again for the purpose of focusing on the main topic of this study, we have simplified the model of perceptual grouping to only include edge segments. As was recently argued by von der Heydt and Zhang [47], the distribution of edges (contours) is sufficient to group features of isolated compact objects but does not disambiguate the assignment of borders between two overlapping objects. It was found in earlier modeling work [25] that addition of local features, in particular T-junctions (physiologically represented by end-stopped cells [58]), can resolve this ambiguity. These model assumptions are strongly supported by recent neurophysiological results showing that even single feature elements, like T or L junctions, strongly influence the firing rates of BOS cells in the case of overlapping figures [47]. The firing rates of BOS cells that represent features of a foreground object are increased by the presence of an L junction that is part of this object, and suppressed by a T junction that is inconsistent with the presence of this object. Remarkably, single occurrences of these local features have a strong effect on BOS cell activity, and additional consistent features only make small additional contributions (*ibid*).

One possibility how local cues influence BOS cell firing rates is by increasing the firing rates of the object-based *G* cells representing foreground objects. The prediction is that the firing rate of the foreground *G* cell is substantially higher than that of the *G* cells representing the objects partially occluded by it. An additional prediction motivated by the data in ref [47] is that *G* cell activity is subject to a strong compressive (saturating) nonlinearity. Another possibility is that neurons responding to local cues directly project to BOS neurons to modify their firing rates [25, 26]. Of course these two possibilities are not mutually exclusive, both could occur. In addition, there are local cues in the spectral domain that can be used to distinguish figure from ground, and therefore border ownership [59, 60]. Incorporating the influence of strong local features in our model as well as the influence of strong saturation is an obvious topic for future work.

### Comparison to previous models and limitations of the present model

Several models have been proposed to account for the mechanism of the modulations of BOS neuronal activity during object perception. Craft et al. [25] developed a computational model of the BOS mechanism based on the hypothetical grouping circuit. Mihalas et al. [26] proposed a computational model for explaining how selective attention that was mediated by top-down projections from *G*-cells modulates the responses of BOS neurons. Russell *et al*. [28] opened the feedback loop between BOS and G cells and implemented an efficient feedforward model that can process arbitrary visual input, including natural scenes and, in later work, video [61]. However, these models mainly demonstrated how top-down selective attention modulates the firing frequency of BOS neurons, without considering neuronal dynamics and spike synchrony. Furthermore, in these models, top-down signals for modulating the activity of BOS neurons are implemented functionally, without regard to the biophysical mechanism how the modulation is achieved. In contrast, this model, as well as its predecessor [41] suggests that the bottom-up input from the visual periphery drives BOS cells by AMPA-type synapses while feedback signals mediating grouping and attention rely on NMDA synapses (we discuss other types of synapses below). An additional major advance of the current study compared to that in ref [41] is that we here add an explicit mechanism for spatial attention.

In our model, modulatory feedback from G-cells is implemented by glutamatergic synapses of the NMDA type and feedforward input representing visual stimuli relies on AMPA type synaptic currents. We show that this combination of synaptic circuitry is sufficient to explain a substantial part of neurophysiological observations. However, it is believed that other neurotransmitters and neuromodulators also play a role in the control and implementation of selective attention, in particular acetylcholine and dopamine [62–64], which we do not consider in this study. Dopamine-mediated activity within the frontal eye field (FEF) may be involved in the determination of saccadic target selection and the modulation of responses of V4 neurons [64]. Signals from FEF modulate responses in visual cortices during tasks requiring spatial attention [65, 66]. It is therefore possible that dopamine plays a role in modulating the neuronal activities in visual areas including V2 where most BOS neurons are located. It was also found that cholinergic modulation participates in bottom-up attention and saliency-based selection in cortex [67] and midbrain [62, 68, 69]. Further studies for synapses mediated by these neuromodulators are necessary for understanding the detailed mechanism of attentional modulation in BOS neurons. The present study focuses on the cortical circuitry (Fig 1) while these modulatory influences originate in subcortical structures which we do not consider here. However, they will need to be included in a more complete model.

Another limitation of our study is that we include only the minimum number of BOS neurons to understand the fundamental mechanism for understanding temporal correlations between BOS neurons. In particular, we do not consider connections between BOS neurons. However, the related Craft *et al*. model did incorporate recurrent connections between excitatory and inhibitory BOS neurons [25].

The Craft *et al*. model also includes recurrent connections between BOS neurons and G-cells which autonomously generate activity patterns that implement the object grouping signals in G cell as well as the observed physiological responses of BOS neurons. Since that study showed that it is possible to construct circuitry with G cell activity that results in BOS cell firing rate pattern consistent with experimental findings, we take the existence of such circuitry as given. We therefore *assume* that the G cells already have the firing rates corresponding to the binding and non-binding conditions, respectively, rather than *building* the circuitry that generates these firing rates. This simplifies our already quite complex simulations and allows us systematically explore the influence of the different G cell types on the BOS cells in the object grouping and attention conditions which are the focus of this study.

Finally, a limitation of our model is that it does not require a role for inhibitory interactions even though are clearly present in cortical circuitry. Synaptic inhibition modulates short-timescale correlations, such as synchrony between groups of excitatory neurons (review: [70]). Previous computational studies suggested that attentional modulation for cortical activities is induced by feedback projections to classes of inhibitory neuron [71, 72]. In contrast, the role of inhibitory neurons is not addressed in our model. As mentioned, however, a previous model [25] that shares many features with the present one does employ recurrent inhibitory interactions between BOS cells.

## Materials and methods

### Model architecture

We extend our previous model [41] to understand the neural mechanisms underlying border ownership selectivity. Fig 2 shows the architecture of our network model. It consists of four border ownership selective cell populations, ($BOS_{R}^{1}$, $BOS_{L}^{1}$, $BOS_{R}^{2}$, and $BOS_{L}^{2}$), and four grouping cell populations ($G_{obj}^{1}$, $G_{obj}^{2}$, $G_{obj}^{3}$, and *G*_*sp*_). Populations of BOS cells project to *G*_*obj*_-cells which, in turn, bias the BOS cells resulting in the BOS responses of the latter. We assume that perceptual organization of a visual scene is represented by the combined activity patterns of *G*-cells and BOS cells. While BOS neurons are observed in cortical area V2 and neighboring areas V1 and V4, we are agnostic of where the (so-far hypothetical) *G*-cells reside. The influence of BOS neurons on *G*-cell activity was modeled and discussed in previous models [25, 26, 28]. For simplifying the model network, and similar to our previous study [41], we are not concerned with the details how the *G*-cells receive their activity as a function of attentional state and stimulus configuration but, instead, assume that their activity patterns have already been established.

In our network model, we include only the neurons and synaptic connections necessary to understand the fundamental mechanism for modulating consistent and inconsistent pairs of physiological BOS cells [39](see also Fig 1A). The arrows to model populations in Fig 2 indicate synaptic connections in our network model. The BOS neuron whose RF is shown by the left black (gray) oval has right (left) side-of-figure preference and is therefore named $BOS_{R}^{1}$ ($BOS_{L}^{1}$). Subscripts “L” and “R” represent their left and right side-of-figure preferences relative to the center object. The retinotopic position is denoted by the superscript “1” for the left oval or “2” for the right. Therefore, the BOS neuron whose RF is shown by the right oval has left (right) side-of-figure preference and is named $BOS_{L}^{2}$ ($BOS_{R}^{2}$). $BOS_{R}^{1}$ and $BOS_{L}^{2}$ neurons with distinct RFs can be excited when a visual object is present in the center of the field in Fig 2 (“bound” condition). We call them preferred neurons because their preferred border ownership is towards an object located between their RFs which makes both of them members of a consistent pair. The BOS preference of neurons $BOS_{L}^{1}$ and $BOS_{R}^{2}$ points away from the center object in the stimulus, we call them non-preferred neurons. Inconsistent pairs have either one or two non-preferred neurons (Fig 1A).

Three types of inputs are applied to these BOS neurons: bottom-up visual input and top-down signals from two distinct types of *G*-cells, object-based grouping (*G*_*obj*_) and spatial grouping (*G*_*sp*_) cells (Fig 2). Bottom-up input arises from visual stimuli. *G*_*obj*_-cells impart grouping structure and mediate object-based attention, whereas *G*_*sp*_-cells implement the influence of spatial attention. According to our previous work [41], all inputs to model BOS neurons were modeled as stochastic random processes with Poisson statistics, where each event stands for an incoming action potential. As shown by the arrows to model populations in Fig 2, bottom-up inputs for visual stimuli and top-down signals from $G_{obj}^{2}$- and $G_{obj}^{3}$-cells are independent processes, whereas the top-down signals from $G_{obj}^{1}$ are common to $BOS_{R}^{1}$ and $BOS_{L}^{2}$ model neurons representing the same object (consistent pair). By contrast, *G*_*sp*_-cell activates all BOS neurons homogeneously, irrespective of their RF location and their border ownership selectivity [73]. The firing rate of a $G_{obj}^{1}$-cell ($\nu_{G_{obj}^{1}}$) in the “bound” condition is higher than in the “unbound” condition (Fig 1B). In the latter situation, two objects (gray shapes) are located left and right in the scene and $G_{obj}^{2}$- and $G_{obj}^{3}$-cells are activated (“unbound” condition). In addition to this *G*-cell activity corresponding to the geometry of the scene, we assume that top-down attention increases the firing rate of the corresponding *G*-cells further, as in the “bound-attended” condition. We describe the rates of *G*-cells in the Numerical experiments section below.

Previous computational studies [25, 26] have that hypothesized receptive field of the *G*_*obj*_-cells have a variety of sizes, so they can respond to objects of different scales in the visual scene. Zhang and von der Heydt have investigated how responses in physiological BOS neurons are modulated depending on object size [20]. By contrast, in the present study, we have focused on one scale only, and assume that the mechanisms are identical at all scales. The same applies to *G*_*sp*_-cells. We hypothesize that populations of *G*_*obj*_- and *G*_*sp*_-cells with various sizes of receptive field for responding to whole object scales exist in visual cortices, which functionally results in the zoom lens model of attention [54] that has been supported by behavioral and neurophysiological studies [74, 75]. The zoom lens model proposes that attention can subtend to as little as a fraction of a degree of angle, and, in the other extreme, can be dilated to an even distribution over the entire visual field, with a concomitant loss of spatial resolution for larger scales. *G*_*obj*_-cells and *G*_*sp*_-cells with appropriately scaled receptive fields may tile the visual scene for representing objects with a variety of sizes.

### Model neurons and synapses

In our model, the BOS neurons are integrate-and-fire neurons as in previous models [41, 76, 77], and described in detail as follows. The dynamics of the subthreshold membrane potential (*V*) of a model BOS neuron are

$$\begin{array}{c} \frac{dV(t)}{dt}=-\frac{V(t)}{\tau_{m}}+\frac{I_{syn}\left(t\right)}{C_{m}} \end{array}$$ (1)

where *τ*_*m*_ is membrane time constant, and *C*_*m*_ is membrane capacitance. Neuronal model parameters are chosen based on our previous model [41]. *I*_*syn*_(*t*) represents the synaptic current that flows into model BOS neurons. It is the sum of three types of inputs: *I*_*vis*_ from bottom-up visual stimuli, $I_{G_{sp}}$ and $I_{G_{obj}}$ for top-down modulatory inputs from spatial grouping (*G*_*sp*_) and object-based grouping (*G*_*obj*_) cells, respectively,

$$\begin{array}{c} I_{syn}\left(t\right)=I_{vis}\left(t\right)+I_{G_{sp}}\left(t\right)+I_{G_{obj}}\left(t\right). \end{array}$$ (2)

According to our previous model [41], bottom-up excitatory postsynaptic currents (*I*_*vis*_) are mediated by glutamatergic receptors of the AMPA type,

$$\begin{array}{c} I_{vis}\left(t\right)=g_{AMPA}\left(V\left(t\right)-V_{E}\right)w_{vis}s_{AMPA}\left(t\right) \end{array}$$ (3)

where *V*_*E*_ = 0 mV is the reversal potential and *w*_*vis*_ represents the excitatory synaptic weight from visual stimuli to model BOS neurons, chosen as *w*_*vis*_ = 140. *V* is the subthreshold membrane potential of a model BOS neuron from Eq 1. The conductance of the fully activated synapse is *g*_*AMPA*_ = 0.104 nS, and the fraction of open channels of AMPA receptors (*s*_*AMPA*_) is,

$$\begin{array}{c} \frac{ds_{AMPA}\left(t\right)}{dt}=-\frac{s_{AMPA}\left(t\right)}{\tau_{AMPA}}+\sum_{k}\delta (t-t_{vis}^{k}) \end{array}$$ (4)

where the postsynaptic decay time constant is *τ*_*AMPA*_ = 2.0 ms. The sum over *k* runs over all spikes originating from orientation-selective neurons responding to the visual stimuli. Each spike is entered as a Dirac delta function, *δ*(*t*), which assumes a nonzero value at the spike times of the visually driven input neurons ($t_{vis}^{k}$), zero elsewhere, and has an integral of unity over any interval that includes $t_{vis}^{k}$.

In our network model (Fig 2), whereas $G_{obj}^{1}$-cells provide common modulatory feedback inputs for the consistent pair ($BOS_{R}^{1}$ and $BOS_{L}^{2}$ neurons), signals from $G_{obj}^{2}$- and $G_{obj}^{3}$-cells are independently projected onto $BOS_{L}^{1}$ and $BOS_{R}^{2}$ neurons. In contrast, signals arising from *G*_*sp*_-cells carrying spatial attention are mediated by homogeneous projections to all model BOS neurons irrespective of their BOS selectivity. We hypothesize that the modulatory feedback takes the form of currents through NMDA receptors [42, 43]. All NMDA receptors have a voltage dependence that is controlled by [*Mg*^2+^] [78], which we assume as [*Mg*^2+^] = 1 mM. A large variety of NMDA receptors are expressed in the mammalian brain, with different physiological properties that depend on the combination of their subunits (for a review, see [79]). The exact properties of NMDA receptors in the visual cortical circuits is unknown. Here, we will use a standard computational model for generic NMDA receptors [76, 80] in which the NMDA receptors mediated synaptic current $I_{G_{obj}}$ and $I_{G_{sp}}$ are defined as follow:

$$\begin{array}{c} I_{G_{fb}}\left(t\right)=\frac{g_{NMDA}\left(V\left(t\right)-V_{E}\right)}{1+\left[Mg^{2+}\right]\exp\left(-V\left(t\right)/V_{0}\right)/3.57}w_{fb}^{BOS}s_{NMDA}\left(t\right) \end{array}$$ (5)

where the subscript *fb* represents the source of the feedback signals, *obj* for *G*_*obj*_- or *sp* for *G*_*sp*_-cells. We use *V*_0_ = 16.13 mV. $w_{obj}^{BOS}$ = 110 and $w_{sp}^{BOS}=w_{obj}^{BOS}/2$ are parameters symbolizing the synaptic weight from *G*_*obj*_- and *G*_*sp*_-cells to BOS neurons, respectively. The synaptic conductance of a fully open NMDA synapse is *g*_*NMDA*_ = 0.327 nS; however, at resting voltage (-70 mV), voltage-dependent *Mg*^2+^ blockage (implemented by the denominator in Eq 5) reduces the conductance by more than a factor of 20, making it much smaller than that of a AMPA synapse.

The fraction of open NMDA channels in a synapse is *s*_*NMDA*_, defined as

$$\begin{array}{c} \frac{ds_{NMDA}\left(t\right)}{dt}=-\frac{s_{NMDA}\left(t\right)}{\tau_{NMDA,decay}}+\alpha x\left(t\right)\left(1-s_{NMDA}\left(t\right)\right) \end{array}$$ (6)

$$\begin{array}{c} \frac{dx(t)}{dt}=-\frac{x(t)}{\tau_{NMDA,rise}}+\sum_{k}\delta (t-t_{fb}^{k}) \end{array}$$ (7)

where *α* = 1/ms. The rise time for NMDA synapse is *τ*_*NMDA*,*rise*_ = 2 ms, and their decay time constant is *τ*_*NMDA*,*decay*_ = 80 ms [80]. As in the description of AMPA synaptic currents in Eq 4, the sum over *k* is over spike time ($t_{fb}^{k}$), which are now the times of spikes occurring in *G*-cells.

### Numerical experiments

In our network model, model BOS neurons integrate bottom-up inputs, representing object borders, with top-down influences mediating the perceptual grouping structure and selective attention (Fig 2). Since the contents of the RFs are identical for all visual inputs considered (see the three configurations in Fig 1B), the bottom-up input has the same statistics in all three conditions, which were modeled as Poisson spike trains with a mean rate of 200Hz. This input should be interpreted as originating from a population of visually responsive neurons rather than from a single neuron. This rate is chosen according to our previous theoretical work [41].

The *G*_*obj*_-cells activity is based on the integration of the responses of BOS neurons and represents the visual scene in terms of objects, thus providing a fast sketch of the location and rough shapes of objects in the scene [25, 26]. In this work, we focus on the interaction of modulatory top-down influences with the driving bottom-up input. As in our previous model [41], we increased the *G*_*obj*_-cell activity in the bound condition and observed the influence of its activity on BOS neurons. Likewise, we increased the activity of *G*-cells to represent attentional selection of the target object and location without being concerned about the source of attentional input.

The activity of *G*_*sp*_- and *G*_*obj*_-cells were simulated as Poisson-distributed spike trains. The rates of *G*-cells for representing stimulus and attention conditions are summarized in Table 1. In the unbound condition (left panel in Fig 1B), we assume that $G_{obj}^{1}$-cell fires spontaneously with a mean frequency of 5Hz. In the presence of an object in its RF, a *G*_*obj*_-cell increases its activity. Therefore, in the unbound-condition, $G_{obj}^{2}$- and $G_{obj}^{3}$-cells are activated with a mean rate of 30Hz for representing objects on each side. If the object is present but not attended (bound-ignored; middle panel on Fig 1B), the firing frequency of $G_{obj}^{1}$-cell increases to 30Hz. In contrast, the activities of non-preferred *G*_*obj*_-cells decrease to 5Hz. In these ignored conditions, *G*_*sp*_ activity is an independent Poisson spike train of 3Hz. If the object is attended (bound-attended condition; right panel on Fig 1B), the $G_{obj}^{1}$-cell receives both bottom-up input from BOS cells and top-down signal from attentional control areas for object-based attention and its firing frequency increases to 60Hz. Attentional activation of $G_{obj}^{1}$-cell decreases the firing rates of $G_{obj}^{2}$- and $G_{obj}^{3}$-cells to 2.5Hz [25, 26]. At the same time, the activity of the *G*_*sp*_-cell increases to 15Hz. Although we will, for simplicity, refer to the activity of a single *G*-cell, the top-down spike train should be understood as activity originating from a population of *G*-cells, just as the bottom-up input is the combined activity from many visually responsive neurons. Note that stimulus and attention conditions are fully described by the firing frequencies of *G*-cells in our model.

We integrated the differential equations using a fourth-order Runge-Kutta algorithm with a time step of 0.1 ms. We simulated 100 trials of a length of 200 sec each, for a total of 20,000 simulated biological seconds per condition. The first 750 ms of simulated results was always discarded to minimize the effect of transients (in analogy to the onset transients that are routinely removed in electrophysiological experiments, including in ref [39]). We also extended the simulation beyond 200 sec by the length of the correlation window to allow computation of the correlation function (see below). The code for the simulations was written in the C programming language (source code available as S1 Code).

### Analysis of spike synchrony between model BOS neurons

We quantified spike synchrony by first dividing time into bins of 1 ms width, each containing either 0 or 1 spike. A spike train was thus transformed into a stochastic process ($S_{j,k}^{i}\left(n\right)$), where *i* is the trial number, *j* and *k* are the identity of the neuron ($BOS_{k}^{j}$; $BOS_{R}^{1}$, $BOS_{L}^{1}$, $BOS_{R}^{2}$, or $BOS_{L}^{2}$), and *n* is the bin index. $S_{j,k}^{i}\left(n\right)$ is then a binary vector in which each component takes on the value of 0 if there is no spike in the interval [*n*, *n* + 1) ms in the $BOS_{k}^{j}$ model neuron during trial *i* or 1 if a spike is present in this interval.

The correlogram between two spike trains $S_{l,m}^{i}\left(n\right)$ and $S_{p,q}^{i}\left(n\right)$ for $BOS_{m}^{l}$ and $BOS_{q}^{p}$ model neurons is the mean of the cross-correlations over all trials. The cross-correlation operator (⊙) is defined as follows:

$$\begin{array}{c} C^{i}\left(\tau \right)=S_{l,m}^{i}⊙S_{p,q}^{i}=\sum_{\mu =1000-wd}^{201000+wd}S_{l,m}^{i}\left(\mu \right)S_{p,q}^{i}\left(\mu +\tau \right) \end{array}$$ (8)

where *wd* = 250 ms is the maximal window of the cross-correlation function, and *τ* is the time lag between spike trains (−*wd* ≤ *τ* ≤ *wd*). Note that the lower bound of the sum results in the removal of the transient onset activity of 750 ms duration.

Changes in firing rate, *e.g*. those produced by attention, will change the correlations. We compensated for any such effects by subtracting the average spike frequency from the neuron spike train for each trial [36]. The mean spike count per bin of spike train of $BOS_{k}^{j}$ in trial *i* was as follows:

$$\begin{array}{c} f_{j,k}^{i}=\frac{1}{\Theta }\sum_{n=1000}^{201000}S_{j,k}^{i}\left(n\right) \end{array}$$ (9)

where Θ = 200 sec is the length of the simulated trials. The cross-correlation function, *CC*(*τ*), was computed as follows:

$$\begin{array}{c} CC^{i}\left(\tau \right)=\frac{1}{\Theta }\left(S_{l,m}^{i}-f_{l,m}^{i}\right)⊙\left(S_{p,q}^{i}-f_{p,q}^{i}\right) \end{array}$$ (10)

and the correlogram, *CCG*(*τ*), was defined as follows:

$$\begin{array}{c} CCG\left(\tau \right)=\langle CC^{i}\left(\tau \right)\rangle_{i} \end{array}$$ (11)

where 〈 〉_*i*_ denotes the average over trial *i*. Since the spike trains are time-density functions (e.g., *counts*/*ms*), the cross-correlation and correlogram have the dimensions of *coincidences*/*s*^2^. Correlograms were smoothed by a Gaussian kernel with *σ* = 4 ms for facilitating comparison with the neurophysiological data [39]. Following that paper, in the Results section of the main text the correlograms were symmetrized according to the following equation:

$$\begin{array}{c} CCG_{syn}\left(\tau \right)=\frac{1}{2}\left[CCG\left(\tau \right)+CCG\left(-\tau \right)\right]. \end{array}$$ (12)

The magnitude of synchrony between model BOS neurons (*M*^*i*^) is represented as the integral of the correlogram (Eq 10) in the range of ±*T*:

$$\begin{array}{c} M^{i}=\sum_{\tau =-T}^{T}CC^{i}\left(\tau \right)\times binsize \end{array}$$ (13)

where binsize = 1 ms. The average magnitude of synchrony over trials (*AM*) is

$$\begin{array}{c} AM=\langle M^{i}\rangle_{i}. \end{array}$$ (14)

Loose synchrony (correlations on the order of tens of milliseconds) was computed using *T* = 40 ms.

### Jitter method for computing tight synchrony

The application of jitter methods was used for testing the hypothesis that neurons operate at or below any specific temporal resolution [50, 51]. In this method, the data from each neuron are divided into bins based on the jitter window, starting at the stimulus onset. Each spike of each neuron is then independently moved to a new location, selected from the uniform distribution on the jitter window to which it belonged in the original data (see also Fig 2 in Amarasingham et al. [50]). In this way, the number of spikes within each bin is preserved in the resampling data. The advantage of this method is that it helps to disambiguate short-term from long-termp correlations in the correlograms. Shorter jitter windows remove more of the long timescale correlation between the neurons (the loose synchrony) while preserving short time-scale correlation (the tight synchrony). In order to compute the tight synchrony, based on the physiological work [39], the influences of spikes outside 20 ms were removed by implementing an interval jitter method.

For each spike train, spikes were jittered in a uniform distribution in disjoint, contiguous jitter window of 20 ms. Whereas the original spike trains were binned in 1 ms bins with a maximum of 1 spike/bin, the jittered spike trains could have as many spike in a bin as were present in each 20 ms jitter window of the original binned spike train. By shifting the spikes to new positions in each 20 ms jitter window, the overall firing rate profile of each trial was preserved at the resolution of the width of jitter window. Repeating this jittering produced a sequence of surrogate spike trains. The cross-correlation of each of the surrogates produced a distribution of correlogram. The mean of this distribution was subtracted from the mean of correlogram of the original spike trains (see also Eq 11). The *r* jittered correlogram were found by taking the trial-wise mean cross-correlation of each jittered spike train $S_{l,m}^{i,r^{*}}$ and $S_{p,q}^{i,r^{*}}$ in trial *i*, as shown in the previous section:

$$\begin{array}{c} J^{r}\left(\tau \right)=\frac{1}{\Theta }\langle S_{l,m}^{i,r^{*}}⊙S_{p,q}^{i,r^{*}}\rangle_{i} \end{array}$$ (15)

where Θ = 200 second is the length of the simulation trials. In this work, we repeated the above process 200 times, creating 200 surrogate data sets (*r* = 200).

The tightened, jitter-correlated correlogram, CCG*, was found by subtracting the mean of the *r* jittered correlogram, 〈*J*^*r*^〉_*r*_, for the amount of overlap, as follows:

$$\begin{array}{c} CCG^{*}\left(\tau \right)=\langle CC^{i}\left(\tau \right)\rangle_{i}-\langle J^{r}\left(\tau \right)\rangle_{r}. \end{array}$$ (16)

We also computed the integral of the tight synchrony (Eq 16) in the range of ±5 ms:

$$\begin{array}{c} M^{*}=\sum_{\tau =-5}^{5}CCG^{*}\left(\tau \right)\times binsize \end{array}$$ (17)

M* defined by Eq 17 implied the index as the magnitude of the tight synchrony. In a manner similar to regular loose synchrony, the spike trains have bin size of 1 ms.

## Supporting information

S1 Fig Noise correlation between model BOS neurons. The gray, black and red bars represent the noise correlation of the Unbound-ignored, Bound-ignored, and Bound-attended conditions, respectively. A: Noise correlation for consistent BOS neurons. Confidence intervals of noise correlation of this pair for the Unbound-ignored, Bound-ignored, and Bound-attended were 0.20 ± 0.06 (*SD* = 0.09), 0.20 ± 0.05 (*SD* = 0.08), and 0.07 ± 0.06 (*SD* = 0.10), respectively. B: Noise correlation for the inconsistent pairs. Confidence intervals of noise correlation of these pairs for the Unbound-ignored, Bound-ignored, and Bound-attended were 0.04 ± 0.03 (*SD* = 0.05), 0.02 ± 0.03 (*SD* = 0.05), and 0.03 ± 0.02 (*SD* = 0.04), respectively. Asterisks indicate significant differences between conditions (** *P* < 0.01, t-test). Error bars indicate SDs.
(TIF)

S2 FigCorrelation and loose synchrony between bottom-up visual inputs to BOS neurons.The gray, black and red lines and bars represent the cross-correlations of the Unbound-ignored, Bound-ignored, and Bound-attended conditions, respectively. A, B: Cross-correlation and loose synchrony between *G*_*sp*_- and $G_{obj}^{1}$-cells. C, D: Cross-correlation and loose synchrony between bottom-up visual inputs to $BOS_{R}^{1}$ and $BOS_{L}^{2}$ neurons (consistent pair). E, F: Cross-correlation and loose synchrony between bottom-up visual inputs to inconsistent pairs. Note that curves in panels A, C, and E are not normalized. Hyphen in panel B indicates no significant difference between conditions (*p* < 0.1, t-test). Shaded areas in panels A, C, and E represent SEM. Error bars in panels B, D, and F indicate SDs.(TIF)

S1 Code The source code for the proposed model (C programming language). (ZIP)
