# Supplementary figures and images for: Analysis of spiking synchrony in visual cortex reveals distinct types of top-down modulation signals for spatial and object-based attention
Source: PLoS Comput Biol. 2021 Mar 25;17(3):e1008829. doi: 10.1371/journal.pcbi.1008829 (PMC8023487; doi:10.1371/journal.pcbi.1008829)

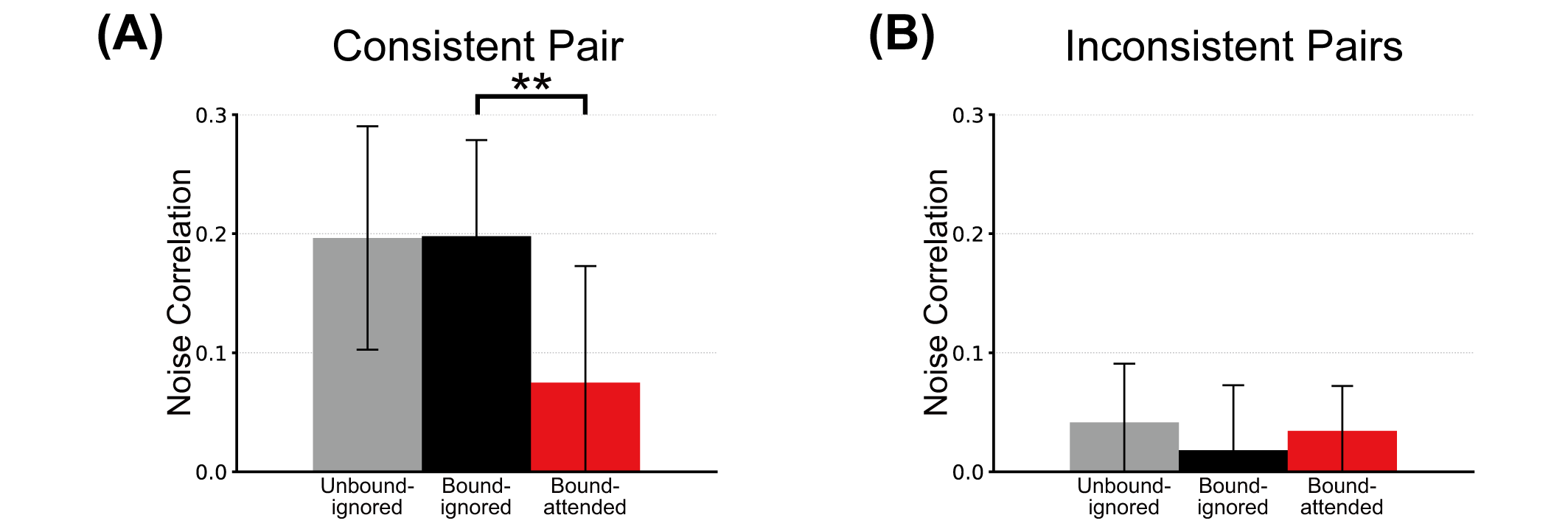

Supplement: S1 Fig — The gray, black and red bars represent the noise correlation of the Unbound-ignored, Bound-ignored, and Bound-attended conditions, respectively. A: Noise correlation for consistent BOS neurons. Confidence intervals of noise correlation of this pair for the Unbound-ignored, Bound-ignored, and Bound-attended were 0.20 ± 0.06 (SD = 0.09), 0.20 ± 0.05 (SD = 0.08), and 0.07 ± 0.06 (SD = 0.10), respectively. B: Noise correlation for the inconsistent pairs. Confidence intervals of noise correlation of these pairs for the Unbound-ignored, Bound-ignored, and Bound-attended were 0.04 ± 0.03 (SD = 0.05), 0.02 ± 0.03 (SD = 0.05), and 0.03 ± 0.02 (SD = 0.04), respectively. Asterisks indicate significant differences between conditions (** P < 0.01, t-test). Error bars indicate SDs. (TIF) [file pcbi.1008829.s001.tif]

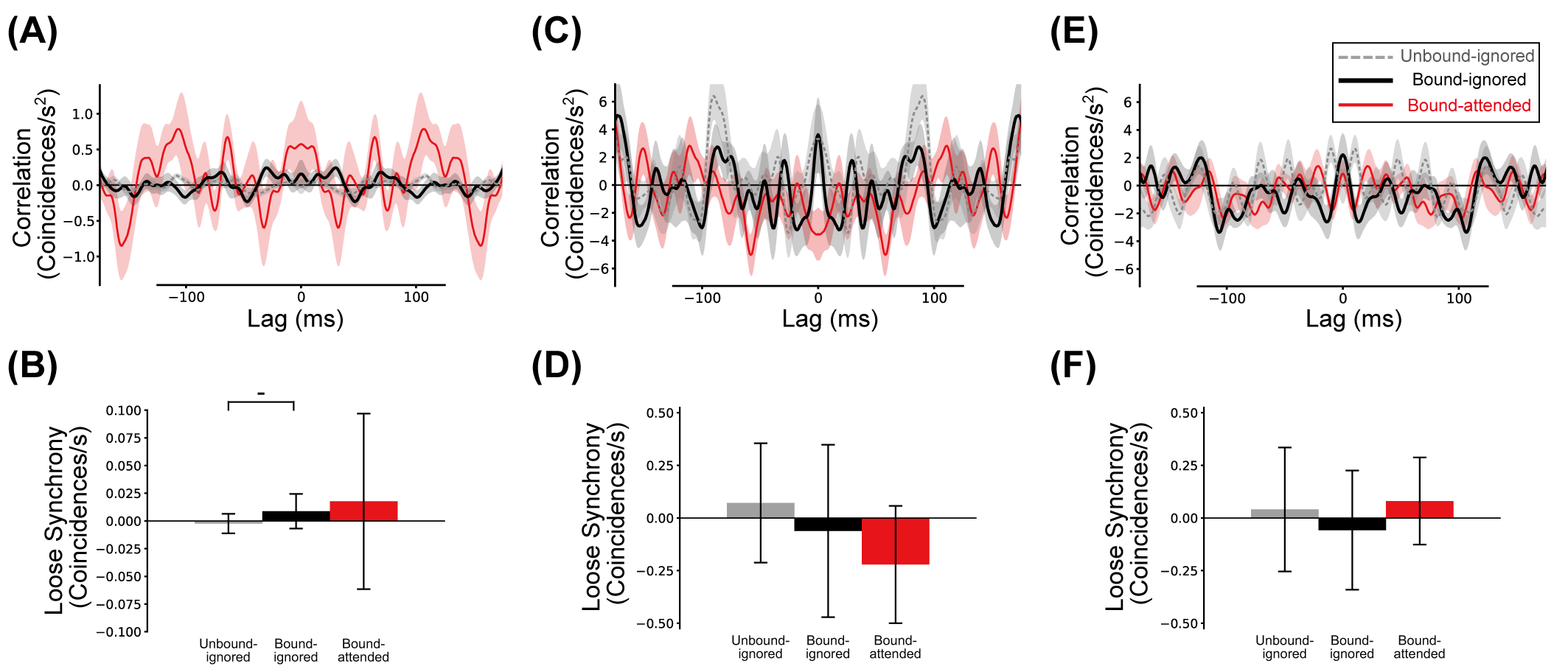

Supplement: S2 Fig — The gray, black and red lines and bars represent the cross-correlations of the Unbound-ignored, Bound-ignored, and Bound-attended conditions, respectively. A, B: Cross-correlation and loose synchrony between Gsp- and Gobj1-cells. C, D: Cross-correlation and loose synchrony between bottom-up visual inputs to BOSR1 and BOSL2 neurons (consistent pair). E, F: Cross-correlation and loose synchrony between bottom-up visual inputs to inconsistent pairs. Note that curves in panels A, C, and E are not normalized. Hyphen in panel B indicates no significant difference between conditions (p < 0.1, t-test). Shaded areas in panels A, C, and E represent SEM. Error bars in panels B, D, and F indicate SDs. (TIF) [file pcbi.1008829.s002.tif]
